# The Neurolipid Atlas: a lipidomics resource for neurodegenerative diseases

**DOI:** 10.1038/s42255-025-01365-z

**Published:** 2025-09-22

**Authors:** Femke M. Feringa, Sascha J. Koppes-den Hertog, Lian Y. Wang, Rico J. E. Derks, Iris Kruijff, Lena Erlebach, Jorin Heijneman, Ricardo Miramontes, Nadine Pömpner, Niek Blomberg, Damien Olivier-Jimenez, Lill Eva Johansen, Alexander J. Cammack, Ashling Giblin, Christina E. Toomey, Indigo V. L. Rose, Hebao Yuan, Michael E. Ward, Adrian M. Isaacs, Martin Kampmann, Deborah Kronenberg-Versteeg, Tammaryn Lashley, Leslie M. Thompson, Alessandro Ori, Yassene Mohammed, Martin Giera, Rik van der Kant

**Affiliations:** 1 Center for Neurogenomics and Cognitive Research, Vrije Universiteit Amsterdam, Amsterdam Neuroscience, Amsterdam, The Netherlands; 2 Alzheimer Center Amsterdam, Department of Neurology, Amsterdam University Medical Center, Amsterdam Neuroscience, Amsterdam, The Netherlands; 3 Leiden University Medical Center, Center for Proteomics and Metabolomics, Leiden, The Netherlands; 4 German Center for Neurodegenerative Diseases (DZNE) Tübingen, Tübingen, Germany; 5 Department of Cellular Neurology, Hertie Institute for Clinical Brain Research, University of Tübingen, Tübingen, Germany; 6 Department of Psychiatry and Human Behavior, University of California, Irvine, Irvine, CA USA; 7 Department of Neurobiology and Behavior, University of California, Irvine, Irvine, CA USA; 8 Institute for Memory Impairments and Neurological Disorders, University of California, Irvine, Irvine, CA USA; 9 Leibniz Institute on Aging, Fritz Lipmann Institute, Jena, Germany; 10 Department of Neurodegenerative Disease, Queen Square Institute of Neurology, University College London, London, UK; 11 UK Dementia Research Institute, University College London, London, UK; 12 Institute of Healthy Ageing, University College London, London, UK; 13 Institute for Neurodegenerative Diseases, University of California, San Francisco, San Francisco, CA USA; 14 Neuroscience Graduate Program, University of California, San Francisco, San Francisco, CA USA; 15 National Institute of Neurological Disorders and Stroke, National Institutes of Health, Bethesda, MD USA; 16 Department of Biochemistry and Biophysics, University of California, San Francisco, San Francisco, CA USA; 17 Gerald Bronfman Department of Oncology, McGill University, Montreal, Quebec Canada; 18 Present Address: Genentech, Inc., San Francisco, CA USA

**Keywords:** Metabolomics, Neuroimmunology, Metabolism, Neuroscience, Lipids

## Abstract

Lipid alterations in the brain have been implicated in many neurodegenerative diseases. To facilitate comparative lipidomic research across brain diseases, we establish a data common named the Neurolipid Atlas that we prepopulated with isogenic induced pluripotent stem cell (iPS cell)-derived lipidomics data for different brain diseases. Additionally, the resource contains lipidomics data of human and mouse brain tissue. Leveraging multiple datasets, we demonstrate that iPS cell-derived neurons, microglia and astrocytes exhibit distinct lipid profiles that recapitulate in vivo lipotypes. Notably, the Alzheimer disease (AD) risk gene ApoE4 drives cholesterol ester (CE) accumulation specifically in human astrocytes and we also observe CE accumulation in whole-brain lipidomics from persons with AD. Multiomics interrogation of iPS cell-derived astrocytes revealed that altered cholesterol metabolism has a major role in astrocyte immune pathways such as the immunoproteasome and major histocompatibility complex class I antigen presentation. Our data commons, available online (https://neurolipidatlas.com/), allows for data deposition by the community and provides a user-friendly tool and knowledge base for a better understanding of lipid dyshomeostasis in neurodegenerative diseases.

## Abstract (web summary)

The authors introduce the Neurolipid Atlas, a dynamic resource for the community to gain insight into lipid alterations in neurodegenerative disease, and they leverage the platform to show how cholesterol alterations in astrocytes can dysregulate neuroinflammatory pathways in Alzheimer disease.

## Main

As one of the most lipid-rich organs in our body^1^, the brain heavily relies on proper lipid homeostasis. Mutations in lipid metabolic genes cause rare but severe juvenile neurodegenerative diseases such as neuronal ceroid lipofuscinoses^2^ and Niemann Pick type C^3^. More recently, changes in lipid metabolism have been implicated in common neurodegenerative diseases such as Alzheimer disease (AD)^4–10^, Parkinson disease (PD)^11,12^, Huntington disease^13–15^, spinocerebellar ataxia^16^, amyotrophic lateral sclerosis (ALS)^17,18^ and frontotemporal dementias (FTDs) including primary tauopathies^19–22^. Conditions associated with neurodegenerative disease pathogenesis such as aging^23^, microglial reactivity to demyelination or fibrillar amyloid-β^24–26^, astrocyte reactivity^27^ and altered sleep cycles^28^ also appear to disrupt brain lipid metabolism.

Together, these findings strongly indicate that alterations in brain lipid metabolism can contribute to neurodegenerative diseases. More importantly, these data suggest that lipid-targeting interventions could be a promising therapeutic strategy to prevent or even treat neurodegenerative conditions. The exact number of endogenous mammalian lipids is unknown. However, it is likely that thousands of individual lipid species together shape cell specific lipidomes (lipotypes) that dictate cellular function and dysfunction in the brain^29^. While standardized data repositories for proteomics and transcriptomics data are common^30–32^, they do not currently exist for (neuro)lipidomics data. As a result of lipid complexity and a lack of standardized tools, sufficient detail on the exact lipid species and downstream pathways that contribute to the various neurodegenerative diseases is lacking. Mapping primary disease-associated changes in the human brain lipidome is particularly challenging, as confounders such as aging, diet, postmortem interval and secondary neurodegenerative processes (for example, cell death) strongly affect lipid metabolism. While animal models have been instrumental for our progress in understanding neurodegeneration, they have limited use for the study of lipids, as the human brain lipidome is intrinsically more complex^33^. Furthermore, while specific brain cell types can be sorted from brain tissue^23,25,34^, studies of lipid metabolism in the human or rodent brain are typically performed in bulk brain tissue, not capturing cell-type-specific changes, for example, in neurons, astrocytes and microglia. The use of induced pluripotent stem cell (iPS cell) technology presents a promising solution to overcome these challenges and enables the study of cell-type-specific regulation of lipid metabolism. Especially in combination with CRISPR–Cas9 gene editing, this technology provides a powerful tool to study how disease-specific mutations and risk variants affect downstream disease phenotypes (for example, amyloid overproduction, pTau levels and α-synuclein levels)^35–38^. Furthermore, iPS cell models are scalable, allowing high-throughput drug discovery of lipid-targeting agents^7^.

To understand genotype–lipid interactions in human brain cells, we developed a standardized pipeline that combines isogenic iPS cell technology and lipidomics analysis capable of quantifying more than 1,000 different lipid species. We also generated a lipidomics data commons, the Neurolipid Atlas (available at https://neurolipidatlas.com/) that allows for user-friendly exploration of (neuro)lipidomics data. For this study, we populated the Neurolipid Atlas with newly generated data from a variety of different human iPS cell-derived disease models and states (AD, PD, ALS and FTDs), as well as novel postmortem human and mouse brain tissue, as benchmarking datasets. Using this pipeline and data analysis tool, we show that human iPS cell-derived neurons (iNeurons), astrocytes (iAstrocytes) and microglia (iMicroglia) have distinct lipid profiles resembling in vivo lipotypes. Through comparative lipidomic profiling of APOE3/3, APOE4/4 and reactive APOE3/3 iAstrocytes, we show that cholesterol esters (CEs) and triacylglycerides (TGs) accumulate in ApoE4 iAstrocytes (as in AD brain) but decrease in reactive astrocytes. Through proteomic and functional characterization, we demonstrate that cholesterol metabolism directly controls astrocyte reactivity, specifically interferon-dependent pathways such as the immunoproteasome and major histocompatibility complex (MHC) class I antigen presentation. High levels of free cholesterol enhance immune reactivity, whereas cholesterol esterification (increased in ApoE4 astrocytes) buffers immune reactivity. Overall, the Neurolipid Atlas provides a neurolipidomics data repository and research tool for the neuroscience field, forming a cornerstone for future research into cell and (neurodegenerative) disease-specific alterations of lipid metabolism. The presented datasets specifically generated for the launch of the Neurolipid Atlas constitute a large open-access collection of neurolipidomics data. Here, we exemplify the potential of our tool by combing multiple lipidomics datasets to show that altered cholesterol metabolism in ApoE4 astrocytes affects their immune reactivity.

## Results

### Lipid profiles of human iNeurons, iAstrocytes and iMicroglia recapitulate known in vivo lipotypes

**Fig. 1:**
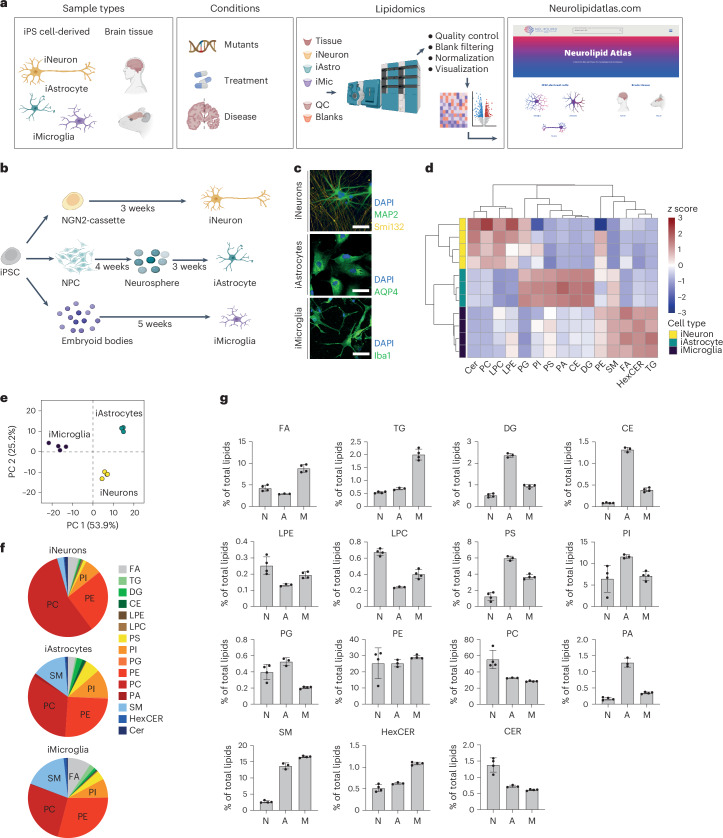
Lipotypes of human iNeurons, iAstrocytes and microglia. **a**, Schematic overview of the Neurolipid Atlas workflow and resource. **b**, Schematic overview of iPS cell differentiation protocols. **c**, Representative confocal microscopy images of iNeurons, iAstrocytes and iMicroglia in monoculture of at least three independent differentiations per cell type. Scale bar, 50 μm (BIONi037-A parental line). **d**, Heat map of *z*-scored lipid class abundance in iNeurons, iAstrocytes and iMicroglia (BIONi037-A parental line). **e**, PCA analysis of iPS cell-derived brain cell lipotypes. **f**, Pie charts showing relative abundance of all detected lipid classes in the iPS cell-derived brain cell types. **g**, Bar graphs present individual lipid class levels in each cell type, normalized to total lipid level. N (iNeurons), *n* = 4 wells; A (iAstrocytes), *n* = 3 wells; M (iMicroglia), *n* = 4 wells. Data are shown as the mean ± s.d. Images in **a**,**b** were created using BioRender.com.

**Extended Data Fig. 1:**
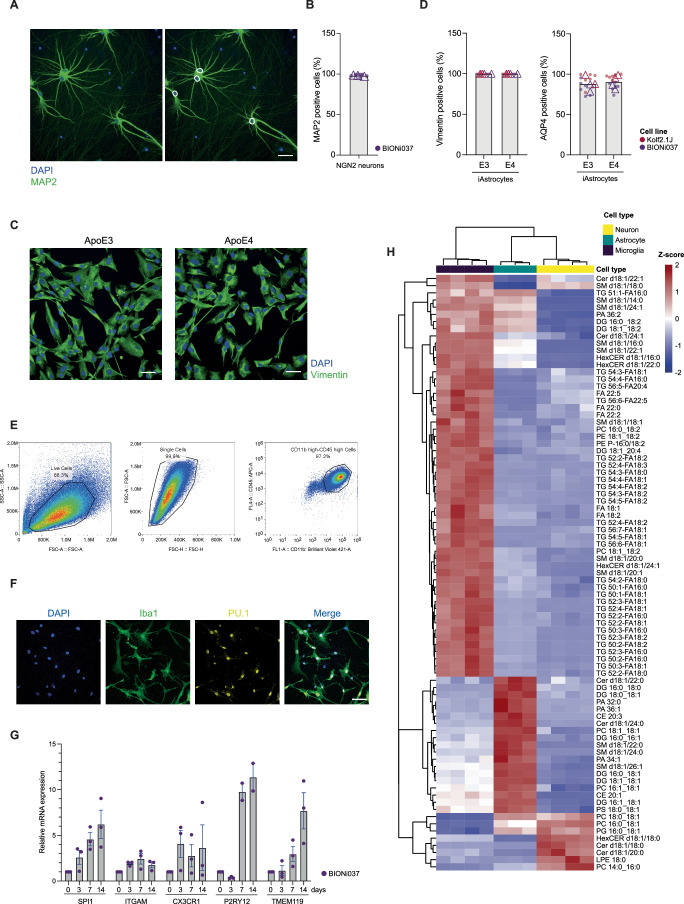
Culture purity and lipotypes (most differentiating species) of human iPSC-derived neurons, astrocytes and microglia. **a**) Representative image of iNeurons used for purity quantification. White circles in the right panel indicate ring region around automatically detected intact nuclei where MAP2 intensity was measured. Scale bar = 50μm (BIONi037-A parental line). **b**) Percentage of MAP2 positive cells. N=3, three independent experiments. Mean **c**-**d**) Representative images (**c**) and quantification (**d**) of the percentage of Vimentin or AQP4 positive ApoE3 and ApoE4 iAstrocytes. N=4 two independent experiments from two isogenic sets. Mean. Scale bar = 50μm. **e**) Representative flow cytometry plots showing that >97% of cells generated from the EB differentiation protocol are pre-macrophages that are then plated for final differentiation to iMicroglia. **f**) Representative images of differentiated iMicroglia (day 14). Scale bar = 50μm (BIONi037-A parental line). **g**) Relative mRNA expression levels determined by qPCR of indicated microglial genes at 0-14 days of differentiation from pre-macrophage stage. N=3, three independent experiments. Mean + sem. **h**) Heatmap of most differentiating lipid species different between iPSC-derived neurons, astrocytes and microglia. alpha = 0.8 is used for discriminant analysis.

To allow easy exploration, analysis and sharing of brain lipidomics data, we generated a novel resource that we named Neurolipid Atlas (Fig. 1a). This resource consists of two modules, for both of which data were newly generated: one module containing datasets generated from iPS cell-derived brain cells and one module containing data from human and mouse whole-brain tissue (Fig. 1a), as discussed below. To understand lipid changes in human brain cells, we developed a standardized iPS cell lipidomics pipeline capable of quantifying >1,000 lipid species across 16 different classes in a cell-type-specific manner (Fig. 1a). iPS cell-derived brain cells have been robustly shown to resemble in vivo brain cell types at the transcript level (albeit being more immature)^39–42^. Whether iNeurons, iAstrocytes and iMicroglia also resemble the in vivo lipidome is not known. Consequently, we differentiated iPS cells from a control iPS cell line (BIONi037-A)^43^ into glutamatergic iNeurons as detailed by Zhang et al.^44^, iAstrocytes as described by Fong et al.^45^ and iMicroglia as described by Haenseler et al.^39^. We confirmed cell fate and purity with cell-type-specific markers MAP2 (neurons), aquaporin 4 (AQP4; astrocytes) and Iba1 (microglia) (Fig. 1b,c and Extended Data Fig. 1a–g). Subsequently, we analyzed the cellular lipidomes using comprehensive, quantitative shotgun lipidomic analysis^46,47^ and found that iNeurons, iAstrocytes and iMicroglia present with distinct lipid profiles (Fig. 1d–g and Supplementary Fig. 1); individual lipid species are listed in Extended Data Fig. 1h. Additionally, we compared the lipidomics data from our iPS cell-derived brain cells to previously published lipotypes of primary mouse cells isolated from brain tissue^48^. Phosphatidylcholine (PC) and phosphatidylethanolamine (PE) were the most abundant lipid class in all cell types. Consistent with mouse brain cells, we observed the highest relative PC and PE levels in iNeurons^48^ (Fig. 1d,f,g). Moreover, the PC-derived and PE-derived lysophospholipids (LPC and LPE) were most abundant in iNeurons. Sphingomyelins (SMs) were highly abundant in iMicroglia, with lower levels in iAstrocytes and very low levels in iNeurons, similar to freshly isolated murine cells^48^ (Fig. 1d,f,g). Consistent with mouse data, phosphatidylserine (PS) was most abundant in iMicroglia and iAstrocytes but low in iNeurons^48^ (Fig. 1d,f,g), while iNeurons had the highest relative levels of ceramides (CERs) (Fig. 1d,f,g). Diacylglyceride (DG) levels were highest in iAstrocytes, as in the published mouse data^48^. Not in keeping with the mouse data were the relatively high DG levels in our iMicroglia, while phosphatidylglycerol (PG) lipids were relatively low (Fig. 1g)^48^. Of the lipid classes that were not measured in the previous mouse study, we found that TGs and free fatty acids (FAs) were most abundant in iMicroglia. iAstrocytes had the highest CE stores, in line with the role of astrocytes as a cholesterol supplier for other brain cell types^49,50^. Overall, these data indicate that iNeurons, iAstrocytes and iMicroglia recapitulate brain cells at not only the transcriptomic and proteomic level but also the lipidomic level. As for the remainder of the manuscript, all lipidomics data are available for exploration, analysis and download through the Neurolipid Atlas (https://neurolipidatlas.com/).

### CEs accumulate in the human sporadic AD brain

**Fig. 2:**
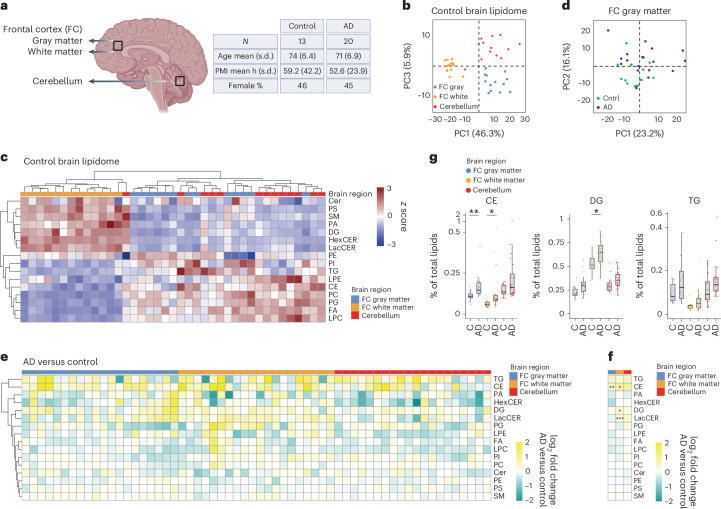
Human (AD) brain lipidomics. **a**, Schematic overview of human postmortem brain tissue samples and summary of participant characteristics. Metadata for individual participants can be found in the Methods. This image was created using BioRender.com. **b**, PCA analysis of unbiased lipidomic analysis from indicated brain areas (control group only). **c**, Heat map shows *z*-scored relative lipid class abundance (control group) per brain region. **d**, PCA plot of unbiased lipidomic analysis of AD (purple) and control (green) brain tissue samples from frontal cortex gray matter. **e**, Heat map depicting changes (log_2_ fold change for AD group versus average control group) at the lipid class level for each individual with AD and each brain area. Samples from participants with AD are ordered 1–20 from left to right in each brain area (metadata in Methods). **f**, Average log_2_ fold change of lipid classes in all AD brain samples compared to control samples per brain area. *P < 0.05 and **P < 0.01 (two-sided *t*-test or Mann–Whitney *U*-test with Benjamini–Hochberg (BH) correction). **g**, Changes in levels of CE, DG and TG (neutral) lipid species in control versus AD group. Data are shown as the median and interquartile range (IQR). *P < 0.05 and **P < 0.01 (two-sided *t*-test or Mann–Whitney *U*-test with BH correction). All lipid values in this figure are plotted as a percentage of total lipids (raw concentration in Extended Data Fig. 2b,f–j).

**Extended Data Fig. 2:**
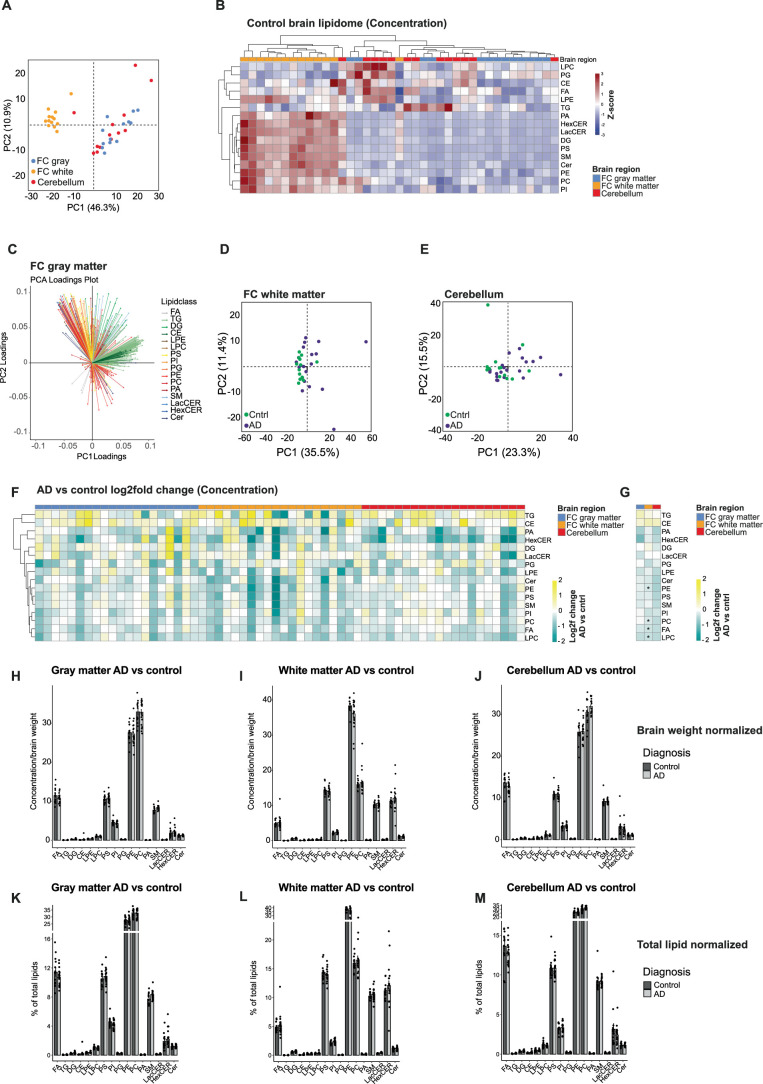
Extended analysis of human (AD) brain lipidomics at the lipid class level. **a**) PCA analysis of unbiased lipidomic analysis from indicated brain areas (control group subjects only, showing PC1 and PC2). **b**) Heatmap with Z-scored lipid class concentrations (nmol/mg brain material) from indicated brain regions (control subjects only, main text Fig. 2c shows same data as percentage of total lipidome). **c**) PCA loadings plot shows how individual lipid species drive PC1 and PC2 in PCA plot of AD vs control brain tissue from Fig. 2d. **d**-**e**) PCA plot of unbiased lipidomic analysis of AD (purple) and control (green) brain tissue samples from (FC) white matter (**d**) and Cerebellum (**e**). **f**) Heatmap depicting changes in lipid classes (based on concentrations) for individual AD samples compared to the average of control samples. Log2fold change plotted independent for each donor and each brain area. **g**) Average log2fold change in AD subject group compared to control samples per lipid class and brain area. Data from (**f**). *P<0.05, t-test or Mann-Whitney U test with Benjamini-Hochberg correction. **h**-**j**) Bar graphs present individual lipid class levels in FC gray matter (**h**), FC white matter (**I**) and cerebellum (**j**) in AD and control samples (group levels) as concentrations. Mean + sd. **k**-**m**) Bar graphs present individual lipid class changes in FC gray matter (**k**), FC white matter (**l**) and Cerebellum (**m**) in AD versus control samples (group level) as percentage of total lipids. Mean + sd.

To compare lipid changes in iPS cell models to those seen in vivo, the second module of the Neurolipid Atlas (Fig. 1a) contains multiple new lipidomic datasets from whole human and mouse brain tissue under different disease conditions. While lipid (specifically cholesterol) changes have been frequently implicated in AD^51^, only few lipidomic studies have been performed on human AD brain tissue^4,52,53^. We determined the control (*N* = 13) and AD (*N* = 20) lipidome across three different brain areas (Fig. 2a). We selected a brain area where AD pathology is abundant (frontal cortex) and an area where pathology is generally lower (cerebellum)^54^. In addition, within the frontal cortex, we differentiated between gray matter (low in myelin) and white matter (rich in myelin). We found significant regional differences in brain lipid composition (Fig. 2b,c and Extended Data Fig. 2a,b). White matter from the frontal cortex had high levels of CERs and SM, consistent with the enrichment of these lipids in myelin (Fig. 2c and Extended Data Fig. 2b)^48,55^. On the contrary, phospholipid and storage lipid (for example, CE and TG) levels were relatively higher in gray matter from the cerebellum and frontal cortex (Fig. 2c and Extended Data Fig. 2b). When analyzing age-matched (Supplementary Figs. 2 and 3) AD versus control brains, principal component analysis (PCA) largely separated control and AD samples in gray and white matter from the frontal cortex but less so in the cerebellum (Fig. 2d and Extended Data Fig. 2c–e). This separation was driven mostly by TG and CE species (Extended Data Fig. 2c). At the class level, we found that CEs were significantly upregulated in AD in gray and white matter from the frontal cortex and trended toward increased levels in the cerebellum (Fig. 2e–g and Extended Data Fig. 2f–m). Analysis at the level of individual lipid species also showed an increase for most CE species (Supplementary Fig. 2a–c) but no single CE specie reached significance, likely reflecting high variation in FA tails of CEs in individuals. In addition, other neutral lipids such as TG (trend in all areas) and DG (significant only for white matter from the frontal cortex) were increased in persons with AD (Fig. 2e–g). Lactosyl-CERs (LacCERs) were also significantly increased in white matter from the AD frontal cortex (Fig. 2e,f). Because astrogliosis is known to be increased in late stages of AD and reactive astrocytes adopt a distinct lipid profile with increased phospholipid saturation^27^, we tested whether saturated phospholipid species were enriched in AD brain tissue. No consistent changes in phospholipid or TG saturation could be observed in the AD brain (Supplementary Fig. 2d,e). Our results in human AD brain, combined with previous findings in persons with AD^4,52,53^, strongly suggest that accumulation of neutral lipids (specifically CE) is a key lipidomic hallmark of AD.

### ApoE4 drives CE accumulation in human iAstrocytes

**Fig. 3:**
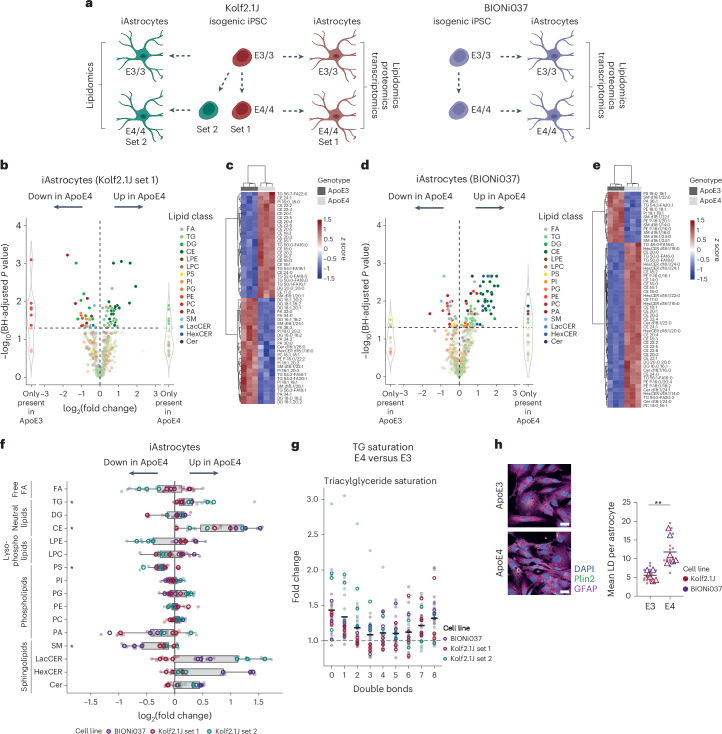
Lipidomic analysis of human isogenic APOE3/3 and APOE4/4 iAstrocytes. **a**, Schematic overview of isogenic iPS cell lines and experimental design. This image was created using BioRender.com. **b**–**e**, Volcano plot presents a typical example of log_2_ fold change of altered lipid species in Kolf2.1J set 1 (**b**) and BIONi037 (**d**) ApoE4 versus ApoE3 iAstrocytes. Also shown are heat maps of most differentiating lipid species between ApoE4 and ApoE3 iAstrocytes in Kolf2.1J set 1 (**c**) and BIONi037 (**e**). **f**, Summary data of changes in all detected lipid classes in ApoE4 versus ApoE3 iAstrocytes (*N* = 9; three independent experiments from three isogenic sets). Data are shown as the median and IQR; whiskers indicate the furthest data point within 1.5× the IQR. *P < 0.05 (two-sided paired *t*-test or Mann–Whitney *U*-test with BH correction). **g**, Fold change in TGs with indicated number of double bonds (unsaturation) in ApoE4 versus ApoE3 iAstrocytes. All experiments presented in **f** are included here. Data are shown as the mean. **h**, Representative images and quantification of the average lipid droplet number per astrocyte based on Plin2 staining (*N* = 6; three independent experiments from two isogenic sets). Data are shown as the mean. **P < 0.01 (two-sided *t*-test). Scale bar, 25 μm. Open circles and triangles indicate the mean per experiment, while solid dots represent all independent wells.

**Extended Data Fig. 3:**
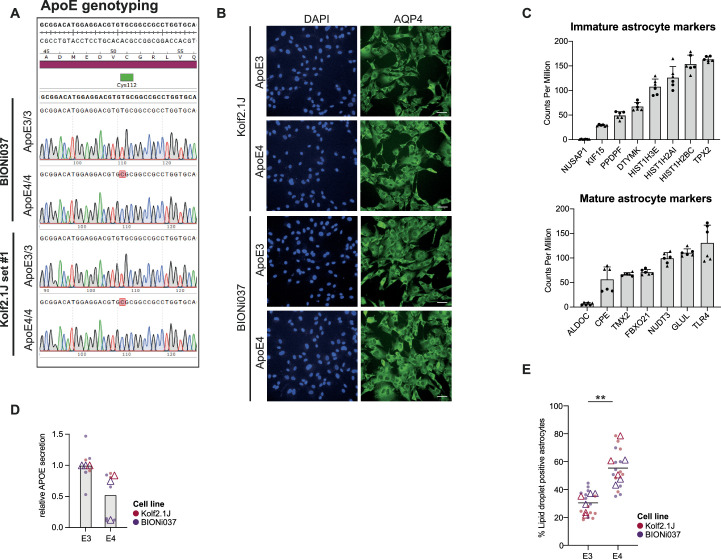
Extended analysis of human isogenic APOE3/3 and APOE4/4 iAstrocytes. **a**) Representative sequencing result for confirming cell identity and expected ApoE genotype. **b**) Representative images of differentiated iAstrocytes, from both Kolf2.1J (set #1) and BIONi037 lines. Scale bar = 50μm. **c**) Gene expression levels (as determined by RNAseq) of indicated mature and immature astrocyte markers in our iPSC-derived astrocytes. n=6 wells BIONi037 (n=3 ApoE3, n=3 ApoE4). **d**) Relative ApoE levels secreted in the medium. N=3, one or two independent experiments from two isogenic sets. Mean **e**) Percentage of LD positive (>5 LDs quantified) iAstrocytes. N=6, three independent experiments from two isogenic sets. Mean **P<0.01 t-test. Data corresponds to Fig. 3h. In all panels open triangles indicate mean per experiment, while solid dots represent all independent wells.

**Extended Data Fig. 4:**
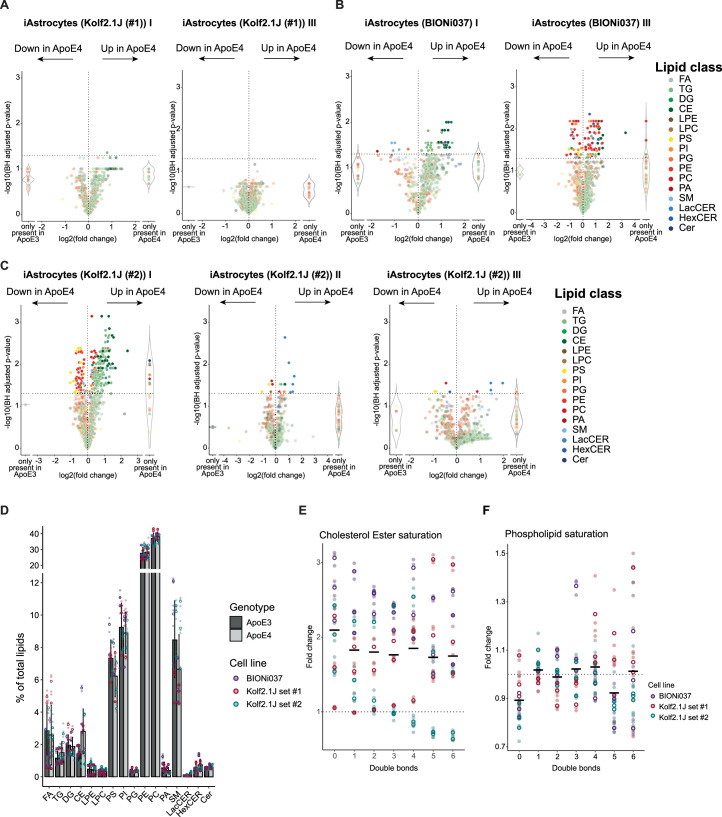
Extended lipidomic analysis of human isogenic APOE3/3 and APOE4/4 iAstrocytes. **a**-**c**) Volcano plots of lipid species changed in ApoE4 vs ApoE3 iAstrocytes in the independent replicate experiments in Kolf2.1J set #1 (**a**), BIONi037 (**b**) or Kolf2.1J set #2 (**c**). **d**) Bar graphs of lipid class levels in ApoE3 and ApoE4 iAstrocytes from all datasets presented in Fig. 3f. N=9, three independent experiments from three isogenic sets, Mean + sd. **e**-**f**) Fold change of cholesterol ester (**e**) or phospholipid (**f**) species with indicated number of double bonds in ApoE4 vs ApoE3 iAstrocytes. Mean. In all panels open circles indicate mean per experiment, while solid dots represent all independent wells.

With CE accumulation being a key lipid feature of AD, it is important to understand how it might contribute to AD pathogenesis. We previously showed that CE accumulation drives pTau buildup in human neurons^7^ and others showed that CE buildup alters microglial function after microglia phagocytose cholesterol-rich myelin^24,25^. While CE levels are highest in human iAstrocytes (Fig. 1), the possible effect of CE accumulation in these cells is unknown. Importantly, astrocytes express high levels of the AD risk gene and cholesterol carrier APOE. A common variant in ApoE, ApoE4, is the major genetic risk factor for AD, which increases the risk for AD from 3–4-fold (heterozygosity) to 14-fold (homozygosity) depending on ethnicity^56,57^, with even higher reported odds ratios in neuropathologically confirmed cases^58^. To map how ApoE4 affects the astrocytic lipidome, we differentiated isogenic pairs of APOE3/3 and APOE4/4 iPS cells to iAstrocytes. We selected isogenic pairs from the iPS cell Neurodegenerative Disease Initiative (iNDI; parental line, Kolf2.1J, APOE3/3; edited lines, APOE4/4 Kolf2.1J C112R hom3 (set 1) and C112R hom2 (set 2))^59,60^ and a completely independent isogenic set from the European Bank for iPS cells (parental line, BIONi037-A, APOE3/3; edited line, BIONi037-A4, APOE4/4)^43^, all from Caucasian origin (Fig. 3a). Neither of these isogenic pairs has been characterized by lipidomic and/or proteomic profiling. The ApoE genotype of the iPS cells was confirmed by Sanger sequencing on receipt and after differentiation to astrocytes, as well as after each experiment to verify sample identity (Extended Data Fig. 3a). Successful differentiation to astrocytes was validated by astrocyte marker staining and evaluation of astrocyte marker expression by RNA sequencing (RNA-seq)^61^ (Extended Data Fig. 1c,d, Extended Data Fig. 3b,c). Less secreted ApoE was present in ApoE4 iAstrocytes (Extended Data Fig. 3d), as has been reported in other iAstrocytes and cerebrospinal fluid (CSF)^5,8,10,62,63^. We performed lipidomics on three isogenic pairs using our standardized iPS cell lipidomic pipeline (individual replicates in Fig. 3b–e and Extended Data Fig. 4a–c; group-level results in Fig. 3f and Extended Data Fig. 4d). Strikingly, consistently across experiments and lines, we observed an ApoE4-dependent increase in CE (Fig. 3f). This effect was mediated by an increase in all detected CE species (Extended Data Fig. 4e). Multiple TG species were also significantly increased, which resulted in a significant increase at the class level (Fig. 3b–f and Extended Data Fig. 4a–d). TGs containing saturated or monounsaturated FAs and highly polyunsaturated FAs (>5 double bonds) were most upregulated in our ApoE4 iAstrocytes (Fig. 3g). Consistent with higher levels of the neutral storage lipids, lipid droplets were increased in ApoE4 iAstrocytes (Fig. 3h and Extended Data Fig. 3e). SM levels were significantly downregulated in ApoE4 iAstrocytes at both the species and the class level (Fig. 3b–f and Extended Data Fig. 4a–d). The BIONi037 ApoE4 iAstrocytes also showed a strong and consistent increase in LacCER, HexCER and CER species, which was not consistently observed in the Kolf2.1J ApoE4 iAstrocytes (Fig. 3b–f and Extended Data Fig. 4a–d). We did not find evidence for increased saturation of phospholipids in our ApoE4 iAstrocytes (Extended Data Fig. 4f), as is typical for reactive astrocytes^27^. Overall, our data indicate that ApoE4 strongly drives the accumulation of CEs and, to a lesser extent, TGs in human iAstrocytes while decreasing SM levels. Lipidomics data of all isogenic pairs and biological replicates from our ApoE4 and ApoE3 iAstrocytes are available on the Neurolipid Atlas.

### ApoE4 decreases MHC class I antigen presentation and immunoproteasome pathways in human iAstrocytes

**Fig. 4:**
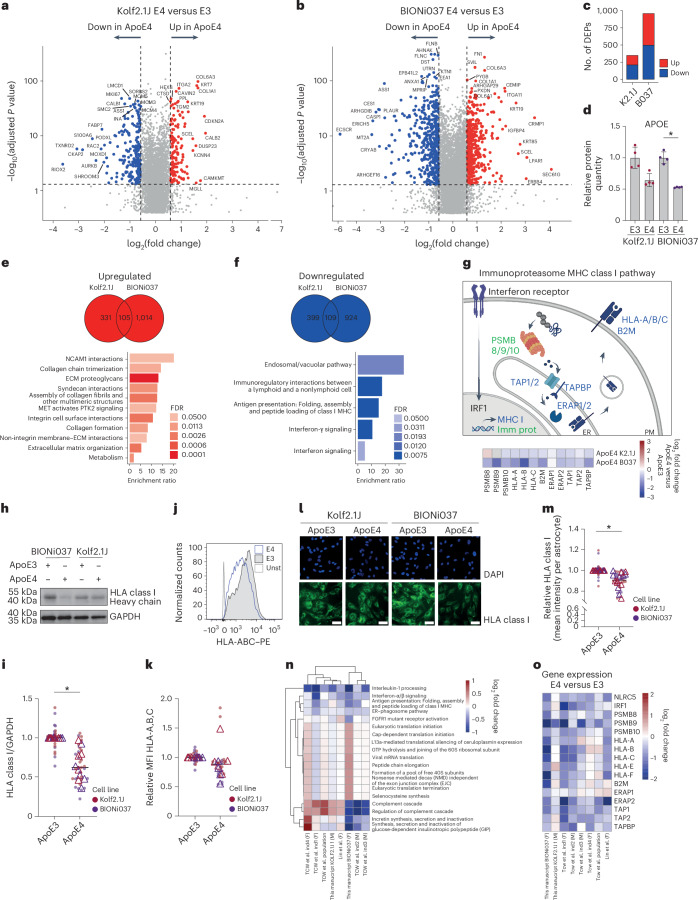
Proteomic and transcriptomic analysis of human isogenic ApoE3 and ApoE4 iAstrocytes. **a**,**b**, Volcano plots present log_2_ fold changes in protein levels in ApoE4 versus ApoE3 iAstrocytes from Kolf2.1J set 1 (**a**) and BIONi037 (**b**). The top ten proteins with the highest log_2_ fold changes and top ten proteins with the most significant *P* values are labeled (*N* = 4 wells per genotype). Statistical analysis was performed using a two-sided pairwise *t*-test. **c**, Number of DEPs (fold change > 1.5, FDR < 0.05) detected in ApoE4 versus ApoE3 iAstrocytes of Kolf2.1J (set 1) and BIONi037 isogenic sets. **d**, Relative ApoE protein levels in ApoE3 and ApoE4 iAstrocytes (from proteomic analysis) from BIONi037 and Kolf2.1J background. Data are shown as the mean and s.d. *P < 0.05 (two-sided Mann–Whitney *U*-test). **e**,**f**, Venn diagrams depicting the number of DEPs significantly upregulated (**e**) or downregulated (**f**) (fold change > 1.25, log_2_ fold change > 0.3) in Kolf2.1J, BIONi037 or both ApoE4 iAstrocytes. A Reactome ORA was performed on the 105 common upregulated (**e**) or 109 common downregulated (**f**) proteins and the enrichment ratio was plotted for all significant pathways (FDR < 0.05). **g**, Schematic overview of interferon-dependent regulation of MHC class I antigen presentation (in blue) and immunoproteasome (in green) pathways. The heat map indicates the log_2_ fold change of indicated proteins in ApoE4 versus ApoE3 iAstrocytes (proteomics). PM, plasma membrane. This image was created using BioRender.com. **h**,**i**, Representative western blot (**h**) and quantification (**i**) of MHC I levels (stained for HLA class I heavy chain) in ApoE4 versus ApoE3 iAstrocytes (*N* = 10; five independent experiments from two isogenic sets). Data are shown as the mean. *P < 0.05 (one-sample *t*-test). **j**,**k**, Representative histogram (BIONi037) (**j**) and quantification (**k**) of plasma membrane MHC I levels (stained for HLA-A, HLA-B and HLA-C) by flow cytometry (*N* = 9; five (Kolf2.1J) or four (Bi037) independent experiments from two isogenic sets). Data are shown as the mean. Unst, unstained control. **l**,**m**, Example images (**l**) and quantification (**m**) of MHC I levels as measured by immunofluorescence microscopy (stained for HLA class I heavy chain) (*N* = 14; seven independent experiments from two isogenic sets). Data are shown as the mean. *P < 0.05 (two-sided one-sample *t*-test). Scale bar, 50 μm. **n**, Comparison of significant Reactome pathways (by gene set enrichment analysis) from our transcriptomic analysis of ApoE4 versus ApoE3 astrocytes (BIONi037) with previously published datasets. Shown is the average log_2_ fold change of all measured genes in the indicated pathway in each isogenic set or case–control set. Tcw et al. ind1–ind4 (four different isogenic sets) and population (control versus ApoE4) represent iAstrocytes from a previous study^10^. Lin et al. represents one isogenic set of ApoE4 versus ApoE3 iAstrocytes from a previous study^8^. F, female; M, male. **o**, Heat map shows the log_2_ fold change in individual genes in the MHC I and immunoproteasome pathway across indicated studies, including our data here. Open triangles indicate the mean per experiment, while solid dots represent all independent wells. Source data

**Extended Data Fig. 5:**
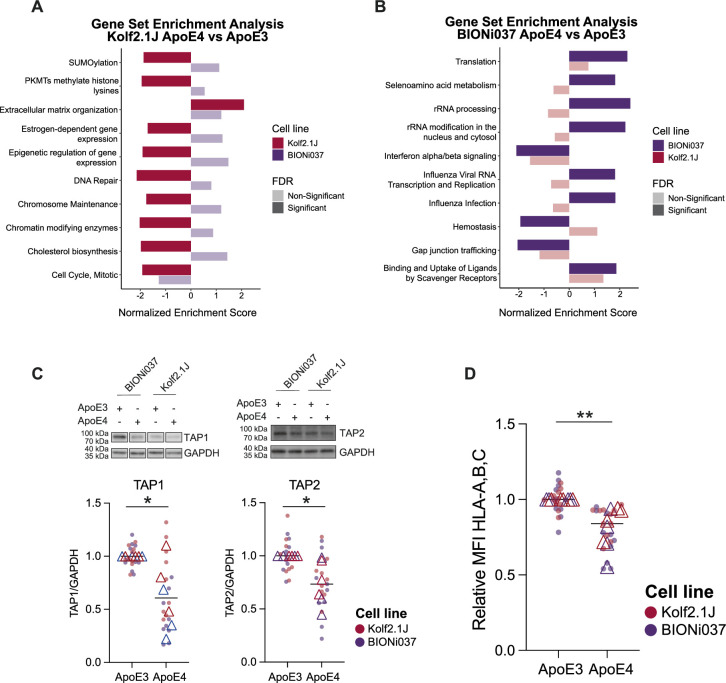
Extended proteomic analysis of human isogenic APOE3/3 and APOE4/4 iAstrocytes. **a**-**b**) Gene-set enrichment analysis with redundancy reduction by a weighted set cover algorithm was performed on proteomics data from ApoE4 versus ApoE3 iAstrocytes for the Kolf2.1J set #1 and the BIONi037 lines. Plotted are the enrichment scores (for both lines) for the Reactome pathways significantly enriched in Kolf2.1J set #1 (**a**) and pathways significantly enriched in BIONi037 (**b**). **c**) Representative western blots and quantifications of TAP1 and TAP2 protein levels in ApoE4 van ApoE3 iAstrocytes. (Kolf2.1J set #1) N=6, three independent experiments from three isogenic sets, Mean *P<0.05 one-sample t-test. **d**) Plasma membrane MHC I levels (stained for HLA-A,B,C) by flow cytometry. Data from Fig. 4k after outlier removal based on the ROUT method (Q=1). Mean **P<0.01 one sample t-test. In all panels open triangles indicate mean per experiment, while solid dots represent all independent wells. Source data

**Extended Data Fig. 6:**
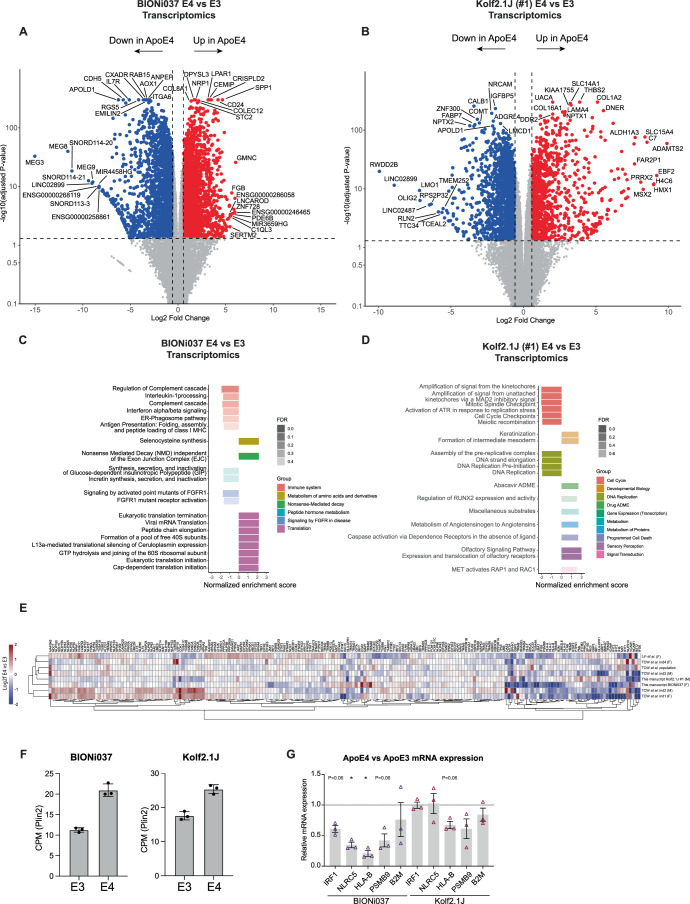
Extended transcriptomic analysis of human isogenic APOE3/3 and APOE4/4 iAstrocytes. **a**-**b**) Volcano plots present log2fold change of differentially expressed genes (DEGs) in ApoE4 vs ApoE3 iAstrocytes from BIONi037 (**a**) or Kolf2.1J set #1 (**b**) from transcriptomics experiments. Top ten genes with the highest log2fold change and top ten genes with the most significant P-value are labeled. **c**-**d**) Top 10 Reactome pathways upregulated or downregulated (with lowest FDR) in ApoE4 vs ApoE3 iAstrocytes by gene-set enrichment analysis of transcriptomics data. **e**) Heatmap shows transcriptomic changes of indicated genes (all genes from the Reactome interferon signaling pathway) in ApoE4 vs ApoE3 iAstrocytes from our study and previous studies (as indicated). **f**) Plin2 gene expression based on the transcriptomic analysis of ApoE4 vs ApoE3 iAstrocytes from BIONi037 or Kolf2.1J set #1. n=3 wells per genotype in each isogenic set. Mean + sd. CPM = normalized counts per million. **g**) Relative mRNA expression levels of indicated genes in ApoE4 vs ApoE3 iAstrocytes quantified by qPCR. N=3 independent experiments from each isogenic set. Mean + sem. *P<0.05 one sample t-test with Benjamini-Hochberg correction on the p-values. (Kolf2.1J set #1).

Our data show that ApoE4 increases CE levels in human iAstrocytes. To understand the functional consequence, we performed proteomic and transcriptomic analysis on our ApoE4 versus ApoE3 iAstrocytes (BIONi037 and Kolf2.1J set 1) (Figs. 3a and 4a–c). Notably, the proteomics and lipidomics were performed on the same batch of iAstrocytes (Methods) to allow for multiomic integration. We found 348 and 959 differentially expressed proteins (DEPs) in Kolf2.1J and BIONi037 ApoE4 iAstrocytes, respectively (Fig. 4a–c and Extended Data Fig. 5a,b). ApoE was among these DEPs, showing downregulation in the ApoE4 iAstrocytes (Fig. 4d). We focused our analysis on proteins that were either downregulated or upregulated in both ApoE4 lines (Fig. 4e,f). Through overrepresentation analysis (ORA), we found that cell adhesion (for example, NCAM1 interactions and integrin cell surface interactions) and extracellular matrix (ECM)-related pathways (for example, collagen chain trimerization and ECM proteoglycans) were upregulated by ApoE4 in both isogenic lines (Fig. 4e). On the contrary, immune pathways (for example, immunoregulatory interactions between a lymphoid and nonlymphoid cell, MHC I antigen presentation and interferon signaling) were downregulated (Fig. 4f). This includes the term ‘endosomal/vacuolar pathway’, which contained mainly MHC terms (Fig. 4f). Strikingly, proteins in the MHC class I antigen presentation pathway were consistently downregulated, as were immunoproteasome subunits, two pathways directly downstream of interferon signaling (Fig. 4g). As a confirmatory readout, we stained against human leukocyte antigens (HLAs) class I and transporters associated with antigen processing 1 (TAP1) and 2 (TAP2) and confirmed downregulation by western blot (Fig. 4h,i and Extended Data Fig. 5c). Decreases in HLA were also confirmed by flow cytometry (Fig. 4j,k and Extended Data Fig. 5d) and immunofluorescence staining (Fig. 4l,m and Supplementary Fig. 4). To be able to compare our results to previous transcriptomic studies with different ApoE4 iAstrocytes^8,10^, we also performed transcriptomic analysis (Extended Data Fig. 6a–f). Through unbiased gene set enrichment analysis, the MHC class I antigen presentation pathway was also found to be downregulated by ApoE4 at the transcriptome level in the BIONi037 isogenic set (Extended Data Fig. 6c), which additionally showed downregulation of interleukin and interferon immune signaling pathways, the complement cascade and endoplasmic reticulum (ER) phagosome transport, whereas translation-related terms were upregulated, as was the lipid droplet marker perilipin 2 (Plin2) (Extended Data Fig. 6c,f). When comparing to transcriptomics from other ApoE4 lines, downregulation of the interferon pathway was consistent in all ApoE4 isogenic and case–control sets from Lin et al.^8^ and Tcw et al.^10^ and trended similarly in our Kolf2.1J set (Fig. 4n,o and Extended Data Fig. 6e,g). Changes in other pathways such as the complement cascade and translation initiation were observed but the direction of change was highly variable across lines (Fig. 4n and Discussion). Overall, these data show that the ApoE4 genotype decreases ApoE levels, drives CE accumulation and inhibits interferon signaling-dependent pathways such as MHC class I antigen presentation and the immunoproteasome in human iAstrocytes.

### Reactive human iAstrocytes decrease CE levels and increase MHC I antigen presentation and immunoproteasome pathways

**Fig. 5:**
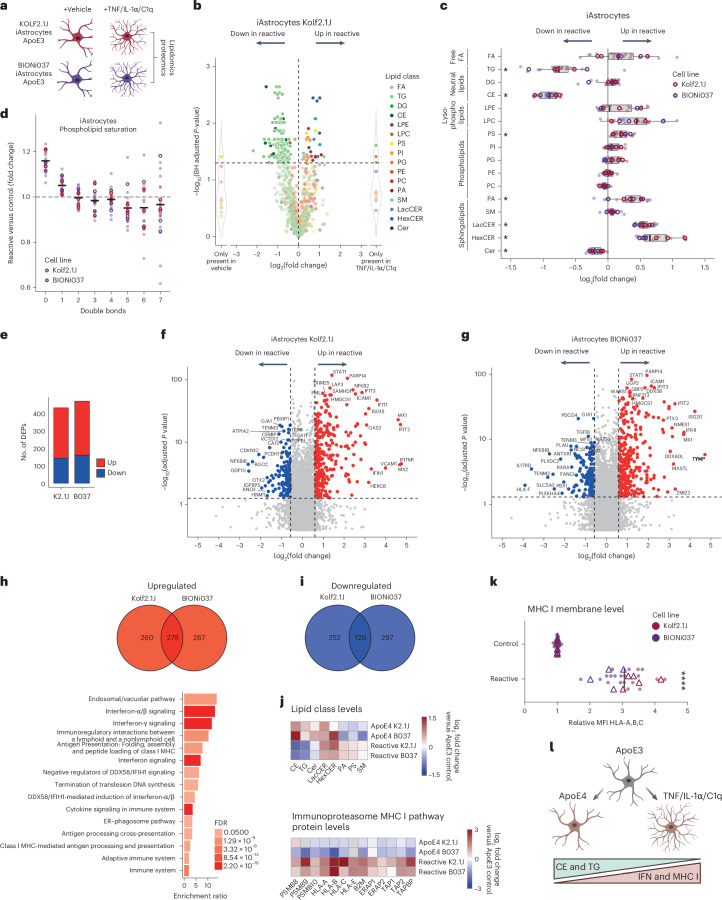
Lipidomic and proteomic analysis of reactive human iAstrocytes. **a**, Schematic overview of experimental design. A cocktail of TNF, IL-1α and C1q was added for 24 h to make astrocytes reactive. **b**, The volcano plot presents the log_2_ fold change of altered individual lipid species in reactive versus control iAstrocytes (Kolf2.1J set 1, ApoE3). **c**, Summary data of changes in all detected lipid classes in reactive versus control iAstrocytes (*N* = 6; three independent experiments from two ApoE3 lines). Data are shown as the median and IQR; whiskers indicate the furthest data point within 1.5× the IQR. *P < 0.05 (two-sided paired *t*-test or paired Mann–Whitney *U*-test with BH correction). **d**, Fold change of all phospholipid species with indicated number of double bonds (unsaturation) in reactive versus control iAstrocytes. All experiments presented in **c** are included here. Data are shown as the mean. **e**, Number of DEPs (fold change > 1.5, FDR < 0.05) in reactive versus control iAstrocytes for indicated lines. **f**,**g**, The log_2_ fold changes in protein levels of reactive versus control iAstrocytes for Kolf2.1J set 1 (**f**) and BIONi037 (**g**). The top ten proteins with the highest log_2_ fold change and top ten proteins with the highest *P* values are labeled (*N* = 4 wells per genotype). Statistical analysis was performed using a two-sided pairwise *t*-test. **h**,**i**, Venn diagram depicting the number of proteins that were significantly upregulated (**h**) or downregulated (**i**) (fold change > 1.25, log_2_ fold change > 0.3) in reactive Kolf2.1J, BIONi037 and both iAstrocytes. A Reactome ORA was performed on the 275 common upregulated or 129 common downregulated proteins. No significantly enriched downregulated pathways were observed; the enrichment ratios for all significantly (FDR < 0.05) upregulated pathways are plotted in **k**. **j**, Top, heat map depicting the log_2_ fold change of indicated lipid classes in ApoE4 or reactive iAstrocytes versus ApoE3 control iAstrocytes. Lipid classes that were changed in ApoE4 or reactive iAstrocytes with *P* < 0.05 are shown. Bottom, heat map depicting the log_2_ fold change of indicated proteins from the MHC class I and immunoproteasome pathway in ApoE4 or reactive iAstrocytes versus ApoE3 control iAstrocytes (based on proteomics data). **k**, Relative membrane MHC I levels (stained for anti-HLA-A, anti-HLA-B and anti-HLA-C) according to flow cytometry in reactive versus control iAstrocytes (*N* = 9; four (Kolf2.1J) or five (Bi037) independent experiments from two isogenic sets). Data are shown as the mean. ****P < 0.0001 (two-sided one-sample *t*-test). **l**, Schematic representation of opposing lipidomic and proteomic phenotypes in ApoE4 and reactive iAstrocytes. Open circles or triangles indicate the mean per experiment, while solid dots represent all independent wells. The images in **a**,**l** were created using BioRender.com.

**Extended Data Fig. 7:**
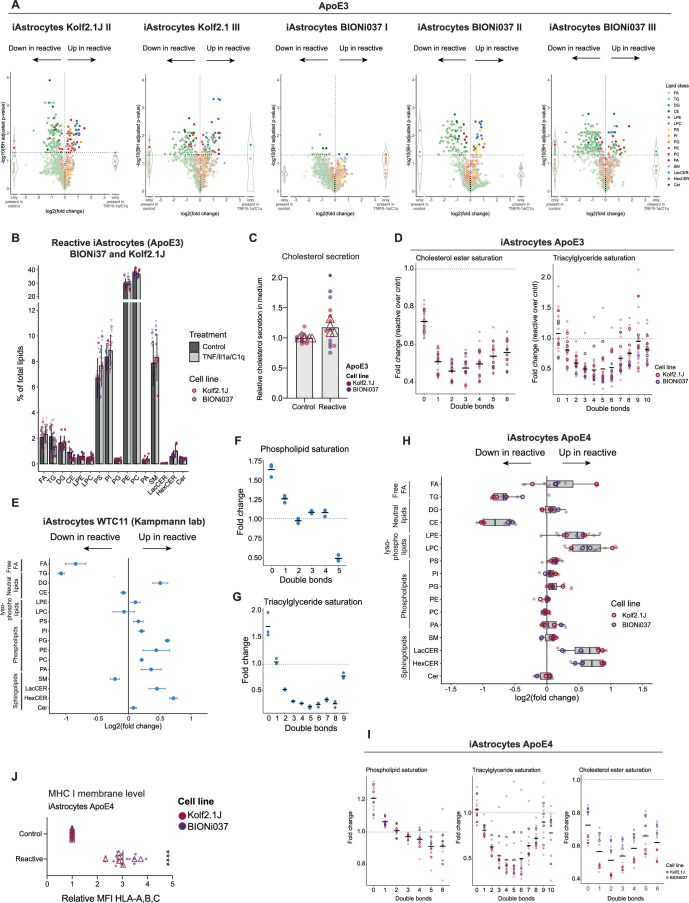
Extended lipidomics analysis of reactive human iAstrocytes. **a**) Volcano plots of lipid species changed in reactive vs control iAstrocytes in independent replicate experiments in Kolf2.1J set #1 ApoE3/3 and BIONi037 ApoE3/3. **b**) Bar graphs of lipid class levels in reactive and control iAstrocytes from all datasets presented in Fig. 5c. N=6, three independent experiments from two ApoE3 lines, Mean + sd. **c**) Relative levels of cholesterol in the medium in control vs reactive iAstrocytes (ApoE3). N=4, two independent experiments from two ApoE3 lines, Mean *P<0.05 one sample t-test. (Kolf2.1J set #1). **d**) Fold change of cholesterol ester (left panel) or triacylglyceride (right panel) species with indicated number of double bonds in reactive vs control iAstrocytes (ApoE3). All experiments presented in Fig. 5c are included here. Mean. **e**) Summary data of changes in all detected lipid classes in reactive vs control iAstrocytes from the Kampmann lab (WTC11 iPSC line, ApoE3/3 cultured in 2% FBS). n=3 wells per condition. **f**-**g**) Fold change of phospholipid (**f**) or triacylglyceride (**g**) species with indicated number of double bonds in reactive vs control iAstrocytes (WTC11, ApoE3). n=3 wells per condition. **h**) Summary data of changes in all detected lipid classes in reactive vs control iAstrocytes (ApoE4). N=4, two independent experiments from two ApoE4 lines. (Kolf2.1J set #1). **i**) Fold change of phospholipid, triacylglyceride or cholesterol ester species with indicated number of double bonds in reactive vs control iAstrocytes (ApoE4). All experiments presented in H are included here. Mean. **j**) Relative membrane MHC I levels (stained for anti-HLA-A,B,C) by flow cytometry in reactive vs control iAstrocytes (ApoE4). N=7, three or four independent experiments from two ApoE4 lines, Mean ****p<0.0001 one sample t-test. (Kolf2.1J set #1). In all panels open triangles or circles indicate mean per experiment, while solid dots represent all independent wells.

**Extended Data Fig. 8:**
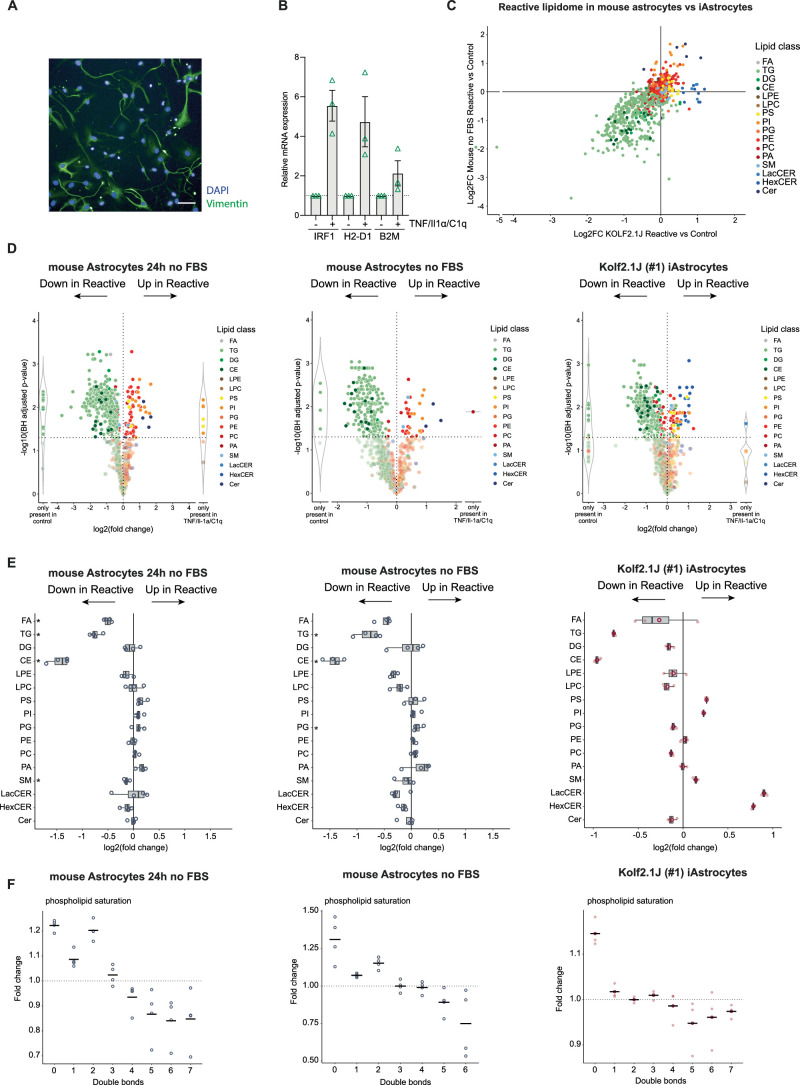
Mouse reactive vs control lipidomics analysis. **a**) Representative image of purified mouse astrocytes. scale bar = 50μm. **b**) Relative mRNA expression of indicated genes in mouse astrocytes after treatment with the reactive cocktail analyzed by qPCR. N=3 mice. Mean + sem. **c**) Scatterplot presents log2fold change of individual lipid species in Kolf2.1J set #1 iAstrocytes vs mouse astrocytes. Both run in the same lipidomics batch to allow for direct comparison. N=4 for mice, n=3 (replicate wells) for iAstrocytes. **d**) Volcano plots present log2fold change of individual lipid species in reactive vs control astrocytes in mouse astrocytes cultured in FBS with 24h FBS removal (as for human astrocytes), mouse astrocytes that never saw FBS and Kolf2.1J iAstrocytes (ApoE3 from set #1) treated with TNF/Il-1α/C1q. N=4 for mice, n=3 (replicate wells) for iAstrocytes. **e**) Summary data of changes in all detected lipid classes in reactive vs control astrocytes of indicated datasets, corresponding to the data shown in D. Median + IQR. *P<0.05 paired t-test or paired Mann Whitney U test with Benjamini Hochberg correction. N=4 for mice, n=3 (replicate wells) for iAstrocytes. **f**) Fold change of phospholipid species with indicated number of double bonds in reactive vs control astrocytes of indicated datasets, corresponding to data shown in D. Mean. In all panels open circles and triangles indicate mean per experiment, while solid dots represent all independent wells.

**Extended Data Fig. 9:**
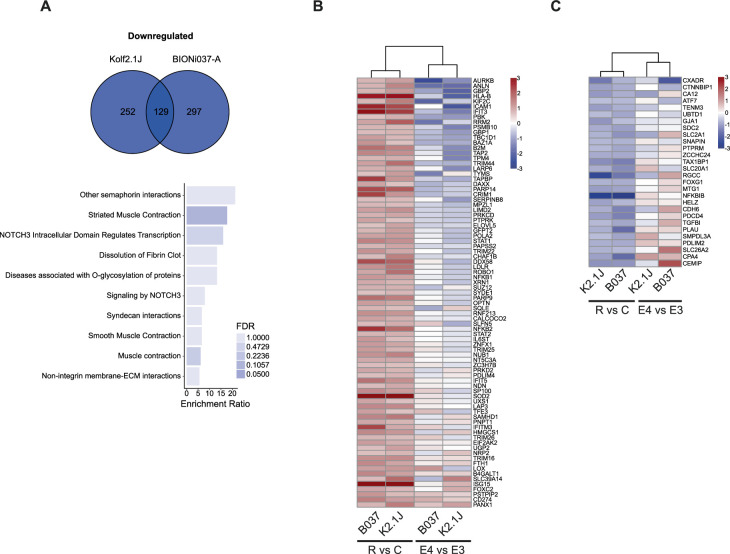
Extended proteomic analysis of reactive human iAstrocytes. **a**) Venn diagram depicting the number of proteins that were significantly downregulated >1.25 fold (>0.3 log2fold) in reactive Kolf2.1J set #1, BIONi037 and both iAstrocytes. Top 10 (non-significant) enriched Reactome pathways detected by overrepresentation analysis are plotted. **b**) Heatmap shows the log2fold change of all proteins significantly upregulated >1.5 fold in both Kolf2.1J and BIONi037 reactive astrocytes. Next to it are the log2fold change values of these proteins in ApoE4 vs ApoE3 iAstrocytes. **c**) Heatmap shows the log2fold change of all proteins significantly downregulated >1.5 fold in Kolf2.1J and BIONi037 reactive astrocytes and the log2fold change values of these proteins in ApoE4 vs ApoE3 iAstrocytes. BIONi037 (B037) and Kolf2.1J (K2.1J).

The reduction in interferon-dependent pathways is striking, as ApoE4 is thought to enhance, not decrease, immune signaling, as astrogliosis is a key hallmark of end-stage AD^5,10,64–67^. Yet, our data clearly demonstrate a consistent reduction in the expression of proteins in these pathways, including five class I leukocyte antigens (HLA-A, HLA-B, HLA-C, HLA-E and HLA-F) and all specific subunits of the immunoproteasome (PSMB8, PSMB9 and PSMB10) (Fig. 4). To better understand our lipidomic and proteomic findings in the context of astrocyte immune function, we also performed multiomic analysis of reactive (treated with tumor necrosis factor (TNF), interleukin-1α (IL-1α) and complement component 1q (C1q)) iAstrocytes (Kolf2.1J and BIONi037-A) (Fig. 5a). By comparing the lipidomic dataset of ApoE4 versus ApoE3 and ApoE3 versus ApoE3 reactive iAstrocytes, we found that neutral lipids were oppositely regulated (Figs. 3f and 5b,c). Whereas CEs and TGs were increased in ApoE4 iAstrocytes, they were strongly downregulated in reactive astrocytes at the species (Fig. 5b and Extended Data Fig. 7a) and class (Fig. 5c and Extended Data Fig. 7b) levels. Cholesterol secretion was increased in reactive iAstrocytes (Extended Data Fig. 7c). Interestingly, LacCER was increased in both reactive iAstrocytes and ApoE4 iAstrocytes, as well as in AD brain (Figs. 2e,f and 5c). Saturated phospholipids were increased in reactive iAstrocytes (Fig. 5d) in contrast to our ApoE4 iAstrocytes (Extended Data Fig. 4f). This increase in saturated phospholipids was also reported in reactive mouse astrocytes^27^. To directly compare how the effect of astrocyte reactivity on the lipidome is conserved across species, we also tested how treatment with TNF, IL-1α and C1q affected the lipidome of mouse astrocytes (under different culture conditions) (Extended Data Fig. 8a–f). In keeping with the human iAstrocytes, reactivity strongly decreased CE and TG levels in mouse astrocytes across culturing paradigms while increasing saturated phospholipid levels (Extended Data Fig. 8c–e). However, the increase in LacCER and HexCER was not observed in mouse astrocytes and, thus, seemed specific to the human astrocytes (Fig. 5b,c and Extended Data Fig. 8c–e). To confirm the effect of reactivity on CE and TG levels (decrease) and LacCER and HexCER levels (increase) in human iAstrocytes, lipidomics was performed on another completely independent parental line (WTC11, M.K. lab) (Extended Data Fig. 7e–g), where the same effect was observed, indicating a robust human astrocyte lipidomic response. While ApoE4 iAstrocytes at baseline have higher CE and TG levels (Fig. 3), stimulation of ApoE4 iAstrocytes with TNF, IL-1α and C1q still reduced CE and TG levels and increased MHC I levels indicating that the ApoE4 effect can be overcome by a strong proinflammatory stimulus (Extended Data Fig. 7h–j). In these lines, an increase in LacCER and HexCER was also observed (Extended Data Fig. 7h). To test whether the decrease in CE and TG lipids in reactive astrocytes was coupled to increased HLA-reactivity, we performed proteomics on the reactive iAstrocytes (Fig. 5e–i). We found 431 and 469 DEPs in Kolf2.1J and BIONi037 reactive iAstrocytes, respectively (Fig. 5e–g). We focused our analysis on proteins that were upregulated or downregulated by reactivity in both lines (Fig. 5h,i and Extended Data Fig. 9a). ORA did not identify any significant downregulated pathways (Extended Data Fig. 9a). However, the top upregulated pathways (Fig. 5h) were the endosome and vacuolar pathway, immunoregulatory interactions between a lymphoid and nonlymphoid cell, MHC I antigen presentation and interferon signaling, pathways that were all downregulated in ApoE4 iAstrocytes (Fig. 4f). Beyond these terms, analysis showed that virtually all immune upregulated proteins in reactive iAstrocytes were downregulated in the ApoE4 versus ApoE3 iAstrocytes at baseline (Fig. 5j, bottom, and Extended Data Fig. 9b,c) and opposite to CE and TG effects (Fig. 5j, top). Using flow cytometry, we confirmed that MHC class I levels (HLA-A, HLA-B and HLA-C) were indeed increased in reactive iAstrocytes (Fig. 5k). Overall, our results indicate that ApoE4 and reactive iAstrocytes have opposing lipidomic and proteomic phenotypes. CEs and TGs are upregulated in ApoE4 astrocytes but downregulated in reactive iAstrocytes, whereas interferon signaling-dependent pathways, the immunoproteasome and MHC class I are downregulated in ApoE4 iAstrocytes but upregulated in reactive iAstrocytes (Fig. 5l).

### Cholesterol metabolism regulates MHC class I presentation and immune reactivity in human iAstrocytes, which is impaired by ApoE4

**Fig. 6:**
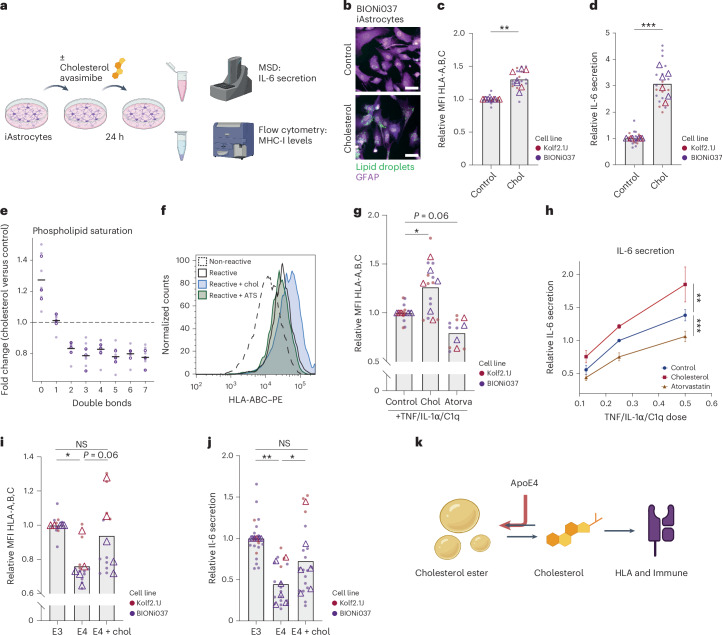
Cholesterol regulates reactivity of human iAstrocytes. **a**, Schematic representation of the experimental design. **b**, Representative image of lipid droplet staining by Lipidspot in iAstrocytes (BIONi037 ApoE3) following 24-h treatment with 50 μM cholesterol. Scale bar, 50 μm. **c**, Normalized membrane MHC I levels (stained for anti-HLA-A, anti-HLA-B and anti-HLA-C) in control versus 50 μM cholesterol-treated ApoE3 iAstrocytes, as determined by flow cytometry (*N* = 7; three (Kolf2.1J) or four (Bi037) independent experiments from two isogenic sets). Data are shown as the mean. **P < 0.01 (two-sided one-sample *t*-test). **d**, Normalized IL-6 secretion in control versus cholesterol-treated ApoE3 iAstrocytes (*N* = 6; two (Kolf2.1J) or four (Bi037) independent experiments from two isogenic sets). Data are shown as the mean. ***P < 0.001 (two-sided one-sample *t*-test). **e**, Fold change of phospholipid species with indicated number of double bonds (unsaturation) in cholesterol-treated versus control iAstrocytes (BIONi037 ApoE3) (*N* = 3 independent experiments from Bi037A iAstrocytes). Data are shown as the mean. **f**,**g**, Representative histogram (**f**) and quantification (**g**) of normalized MHC I membrane levels determined by flow cytometry (stained for anti-HLA-A, anti-HLA-B and anti-HLA-C) in response to indicated treatment conditions in iAstrocytes (*N* = 4–6; three (control and cholesterol) or two (atorvastatin) independent experiments from two isogenic sets. Data are shown as the mean. *P < 0.05 (two-sided one-sample *t*-test for cholesterol and atorvastatin versus 1). **h**, Secreted Il-6 levels in medium of ApoE3 iAstrocytes that were pretreated with or without exogenous cholesterol (10 μM) or atorvastatin (0.5 μM) for 1 h before 24-h cotreatment with increasing doses of TNF, IL-1α and C1q (*N* = 5; two (Kolf2.1J) or three (Bi037) independent experiments from two isogenic sets). Data are shown as the mean and s.e.m. **P < 0.01 (intercept difference by linear regression model). Relative IL-6 levels with vehicle 0.25× cocktail dose set at 1. **i**, Relative changes in membrane MHC I levels determined by flow cytometry (stained for anti-HLA-A, anti-HLA-B and anti-HLA-C) in ApoE3, ApoE4 or ApoE4 iAstrocytes treated with 50 μM cholesterol (*N* = 5; two (Kolf2.1J) or three (Bi037) independent experiments from two isogenic sets. Data are shown as the mean. *P < 0.05 (two-sided one-sample *t*-test for E4 and E4 + cholesterol versus 1). *P < 0.05 (paired *t*-test for E4 versus E4 + cholesterol). BH correction was applied to the three *P* values. NS, not significant. **j**, Relative changes in IL-6 secretion in ApoE3, ApoE4 or ApoE4 iAstrocytes treated with 50 μM cholesterol (*N* = 6; one (Kolf2.1J) or five (Bi037) independent experiments from two isogenic sets). Data are shown as the mean. *P < 0.05 (two-sided one-sample *t*-test for E4 and E4 + cholesterol versus 1). *P < 0.05 (paired *t*-test for E4 versus E4 + cholesterol). BH correction was applied to the three *P* values. **k**, Schematic representation of ApoE4 decreasing MHC class I expression and immune function in human glia by increased cholesterol storage in CEs. Open circles or triangles indicate mean per experiment, while solid dots represent all independent wells. Images in **a**,**k** were created using BioRender.com.

**Extended Data Fig. 10:**
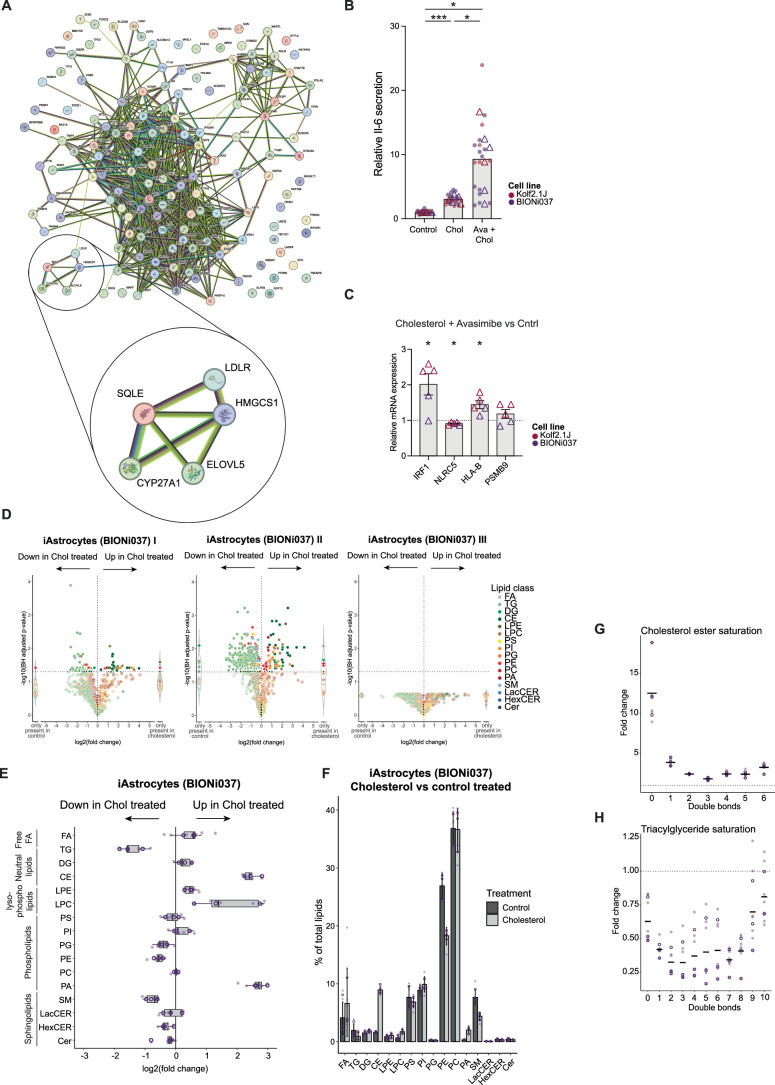
Cholesterol regulates astrocyte reactivity extended data. **a**) STRING analysis of all proteins significantly upregulated >1.5 fold in Kolf2.1J and BIONi037 reactive astrocytes (dataset from proteomics in Fig. 5). Zoom in shows cluster of proteins related to cholesterol metabolism. **b**) Normalized Il-6 secretion in iAstrocytes (ApoE3), pre-treated with vehicle or avasimibe (0.5μM) for one hour and then treated for 24 hours with vehicle, cholesterol (50μM) or cholesterol + avasimibe. N=6, four or two independent experiments from two ApoE3 lines, Mean *P<0.05, ***P<0.001 one sample t-test for Chol and Ava + Chol vs 1. *P<0.05 t-test for Chol vs Ava + Chol with Benjamini-Hochberg correction on the three p-values. **c**) Relative mRNA expression of indicated genes after cholesterol (10μM) + avasimibe (0.5μM) treatment vs control, quantified by qPCR. N=5, two or three independent experiments from two ApoE3 lines, Mean + sem *P<0.05 one sample t-test with Benjamini-Hochberg correction on the p-values. **d**) Volcano plots represent log2fold change of individual lipid species in cholesterol treated vs control iAstrocytes in three independent replicate lipidomics experiments (BIONi037-A). **e**) Summary data of changes in all detected lipid classes in cholesterol vs control treated iAstrocytes (BIONi037-A). N=3 Median + IQR. Paired t-test or paired Mann Whitney U test with Benjamini Hochberg correction. **f**) Bar graphs of lipid classes in cholesterol vs control treated iAstrocytes from BIONi037-A background (% of total). All experiments presented in E are included here. Mean + sd. **g**-**h**) Fold change of phospholipid (**g**) and triacylglyceride (**h**) species with indicated number of double bonds in cholesterol treated vs control iAstrocytes (BIONi037-A). All experiments presented in E are included here. Mean. In all panels open circles or triangles indicate mean per experiment, while solid dots represent all independent wells.

On the basis of the reduction in CEs in reactive iAstrocytes but increase in ApoE4 iAstrocytes, we hypothesized that changes in cholesterol metabolism might directly contribute to immune phenotypes. Using proteomic analysis, we also found that a specific cluster of cholesterol synthesis genes were upregulated in reactive iAstrocytes (Extended Data Fig. 10a). To test whether cholesterol regulates immune reactivity in human iAstrocytes, we treated Kolf2.1J and BIONi037 control (ApoE3) iAstrocytes with cholesterol (Fig. 6a–e). Both MHC class I presentation and IL-6 secretion were significantly increased after cholesterol treatment (Fig. 6c,d). CEs are generated through conjugation of free cholesterol to an FA by acyl coenzyme A cholesterol acyltransferases (ACATs). The combined addition of cholesterol with the ACAT inhibitor avasimibe further increased IL-6 secretion, indicating that CE formation buffered the immune reactivity mediated by cholesterol treatment (Extended Data Fig. 10b). We also found that RNA levels of interferon regulatory factor 1 (IRF1), a master regulator of MHC class I pathway gene expression, and HLA-B were upregulated by exogenous cholesterol (Extended Data Fig. 10c), indicating that the effect of cholesterol on these pathways might be transcriptionally mediated. Moreover, the addition of cholesterol to astrocytes was sufficient to increase saturated phospholipid levels indicative of astrocyte reactivity (Fig. 6e and Extended Data Fig. 10d–h). Exogenous cholesterol also potentiated immune reactivity (as measured by MHC class I levels and IL-6 secretion) of iAstrocytes treated with TNF, IL-1α and C1q (Fig. 6f–h). Conversely, pretreatment with atorvastatin (to reduce cholesterol levels) inhibited MHC class I upregulation and IL-6 secretion upon iAstrocyte reactivity (Fig. 6f–h). Lastly, we found that the addition of free cholesterol rescued MHC I expression and IL-6 secretion in the ApoE4 iAstrocytes (Fig. 6i,j). Overall, our data indicate that cholesterol is a major regulator of MHC class I antigen presentation and immune reactivity in human astrocytes and dysregulated cholesterol metabolism by ApoE4 impairs astrocyte immune reactivity (Fig. 6k).

### The Neurolipid Atlas: an open-access lipidomics data commons for neurodegenerative diseases

**Fig. 7:**
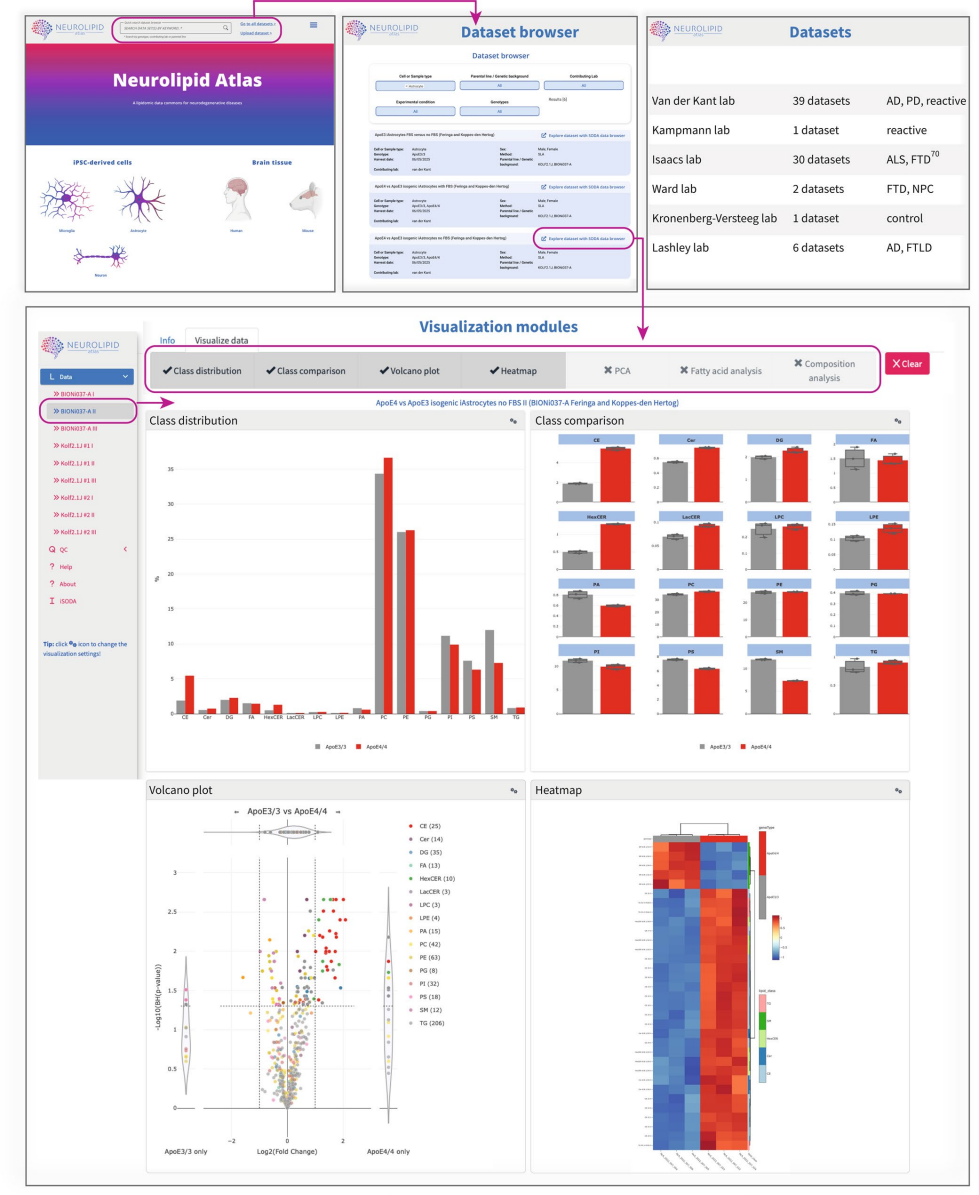
The Neurolipid Atlas. Overview of the Neurolipid Atlas data commons (https://neurolipidatlas.com) to explore all lipidomics datasets from this study. A representative image of the homepage is shown, where one can proceed to a list of experiments by selecting one of the cell type, human or mouse icons or enter a search term as indicated in the top panel. Alternatively, a list of all datasets can be found by selecting; ‘go to all datasets. A link to the quick start guide can be found at the bottom of the homepage or behind the menu (≡) icon in the top right corner. To explore data, experiments can be selected in the dataset browser window as indicated. Independent replicates of the experiment can be selected in the left column under ‘data’. Visualization options can be (de)selected in the top horizontal bar. Examples of bar graphs, volcano plots and heat maps for visualization of changes in lipid class (bar graphs) or lipid species (volcano plots and heat maps) levels between selected conditions are shown. The ‘QC’ and ‘help’ modules present in the left column offer extensive background information on the QC, data-processing steps, lipid class measurements and visualization options. Lastly, a summary list of currently available datasets is shown.

While repositories for RNA and proteomics datasets are common in the field of neurodegenerative diseases, similar resources are currently lacking for (neuro)lipidomics data. To standardize and allow exploration of data without prior bioinformatics knowledge, we generated an online lipidomics browser (Fig. 7), Neurolipid Atlas (https://neurolipidatlas.com/). This browser includes all data presented above, as well as (un)published lipidomics data generated together with a large group of collaborators, currently totaling more than 70 datasets over four neurodegenerative diseases and multiple treatment conditions (Supplementary Table 1). All current datasets in the Neurolipid Atlas (and in the figures of the manuscript) were newly generated for this manuscript, except for the ALS datasets that were recently published^18^. New datasets from our lab and our collaborators will be uploaded to the database in a continuous manner. Labs can contribute their neurolipidomics data to our resource (‘upload dataset’) by providing annotated lipidomics data in line with LipidMaps shorthand notation^68^, a metadata sheet (methods provided on the website) and a short description of the dataset. All data will be quality-controlled (QCed) and curated by the Neurolipid Atlas team, including checking compliance with Lipidomics Standards Initiative^69^, before it is published online. All datasets require a digital object identifier (DOI) from the associated preprint or publication or (for unpublished data) a DOI generated through the lipidomics minimal reporting checklist^69^. The Neurolipid Atlas currently includes Lipidyzer data analyzed by Shotgun Lipidomics Assistant (SLA) software^47^ but other pipelines will also be accommodated. All neuro-related data are welcomed, including iPS cell models, as well as human and animal model brains and cerebrospinal fluid. Contact information, including for information on data uploads, can be found online (https://neurolipidatlas.com/). The Neurolipid Atlas allows for the download of all raw data and metadata, as well as in-browser analysis, which includes QC, blank filtering, normalization and the generation and customization of figures. A link to all datasets is included on the homepage. Datasets can be searched for by name, cell type, genotype, treatment type, parental line or contributing lab. Using the ‘explore dataset with SODA data browser’ link, users can explore independent replicate experiments and visualize changes at the lipid class level (bar graphs) or at the species level (volcano plots, heat maps, PCA and FA analysis) and interact with the data by hovering over different lipid species. All figures and their source data can be downloaded. A comprehensive ‘quick start’ guide is available on the Neurolipid Atlas homepage (https://neurolipidatlas.com/) to help users navigate the data, while a detailed manual outlining all analysis and visualization options is included with each dataset in the help section.

## Discussion

Lipid metabolism is affected and implicated in various neurodegenerative diseases^2–4,6–12,14–20,22,24,25^. Here, we generated a new resource, the Neurolipid Atlas, to facilitate insights into lipid changes associated with different neurodegenerative diseases in a disease-specific and cell-type-specific manner. Leveraging multiple datasets, we demonstrate that iNeurons, iMicroglia and iAstrocytes exhibit distinct lipid profiles that recapitulate in vivo lipotypes. Notably, the AD risk gene ApoE4 drives CE accumulation in human iAstrocytes and CE accumulation is also observed in the human AD brain. Subsequently, through an integrated multiomics approach, we show that CE accumulation in ApoE4 iAstrocytes represses their immune function, specifically interferon pathways, MHC class I antigen presentation and immunoproteasome pathways.

### CE accumulation as a hallmark of AD

Our findings further consolidate the notion that CE accumulation is a major pathogenic hallmark of AD^4,52,53^. CEs in CSF were shown to correlate with progression from mild cognitive impairment to AD^70^. CE accumulation in neurons drives pTau accumulation and Aβ overproduction^7,71–73^. Accumulation of CEs has also been detected in microglia upon TREM2 or ApoE loss^25^ and inhibition of CE formation improved Aβ clearance^74^. In ApoE4 oligodendrocytes, CE accumulation led to perturbed neuronal myelination^75^. We show that ApoE4 iAstrocytes exhibit increased CE accumulation and this altered cholesterol metabolism impairs their immune function, including suppression of the MHC class I pathway. Our data show that CEs accumulate in both white and gray matter of the frontal cortex, which indicates that cells other than solely myelinating oligodendrocytes^75^ contribute to this phenotype. Secondary neurodegenerative processes (protein aggregation, neuronal death and demyelination) and CE accumulation in microglia might be a possible explanation for the observed CE accumulation in persons with AD^24–26^. Yet, our finding that the AD genetic risk factor ApoE4 strongly drives CE accumulation in astrocytes in the absence of pathology indicates that CE accumulation might not merely be a downstream effect of neurodegeneration but rather directly downstream of AD risk genes. How CEs accumulate in ApoE4 astrocytes needs further investigation but could involve altered import and export of cholesterol or changes in lysosomal lipid clearance^76^. Increases in TG levels were reported in the aging mouse brain^23^ and we and others^9^ also observed an increase in TGs in ApoE4 iAstrocytes and a trend toward increased TG levels in the AD brain (Fig. 2).

We confirm previous reports that ApoE4 increases levels of polyunsaturated TGs^9,77^ but also find an increase in monounsaturated and saturated TG levels (Fig. 3g). Importantly, ApoE4 iAstrocytes do not show the increase in saturated phospholipids typical for reactive astrocytes^27^ (Extended Data Fig. 4f). Overall, our data show that ApoE4 and AD present a unique lipotype that is primarily characterized by strong CE accumulation and changes in TGs and SM. To better understand how CE contributes to AD pathogenesis in vivo (through astrocyte immune repression and/or in other cell types), it will be important to define which cell types accumulate CE and how this accumulation potentially alters their function.

### ApoE4 lipid changes and immune suppression

As the strongest genetic risk factor for AD (ApoE) is highly expressed in astrocytes, there is an urgent need to understand how the AD risk variant ApoE4 affects astrocytes. Here, we provide a characterization of isogenic APOE3/3 and APOE4/4 iAstrocytes from the iNDI line Kolf2.1J, which we hope will serve as a reference for the field. We also provide a full characterization of a second isogenic pair of iAstrocytes (BIONi037, EBISC). As astrogliosis is a major feature of end-stage AD^78–81^, we were very surprised to find major immune pathways such as interferon signaling, the immunoproteasome and MHC class I antigen presentation to be downregulated in ApoE4 iAstrocytes. To place this in context, we also generated the first lipidomic and proteomic analysis of reactive human iAstrocytes treated with TNF, IL-1α and C1q. We confirmed that reactive human iAstrocytes, as in mice^27^, also increase phospholipid saturation (Extended Data Fig. 8). Strikingly, the top upregulated proteomic pathways in our reactive iAstrocytes were interferon pathways including MHC class I. Furthermore, CE and TG were downregulated in reactive astrocytes but upregulated in ApoE4 astrocytes (Fig. 5j). The results provide strong evidence that ApoE4 intrinsically inhibits rather than activates astrocyte immune function. These results fit with recent reports in AD mice showing decreased immune function of ApoE4 microglia, including reduced antigen presentation^82,83^. Interestingly, human stem-cell-derived microglia xenotransplanted into *APP*^NL-G-F^ mice were shown to transition into a human-specific HLA-expressing state and ApoE4 selectively reduced the proportion of cells acquiring this HLA phenotype^84^. In line with this, an AD-protective variant in PLCg2 (PLCg2-P522R) was recently shown to reduce CE accumulation in iMicroglia^24^ while increasing microglial MHC I levels and providing benefit through increased recruitment of T cells^85^. On the basis of these data, the presence of a similar ApoE4–cholesterol–immune axis in microglia, as we identified here for astrocytes, is likely but needs to be confirmed. Overall, these findings (including ours in human brain cells) indicate that ApoE4 intrinsically limits immune reactivity rather than inducing immune activation. This could indicate that the immune reactivity (of astrocytes and microglia) is needed to stave off AD and restoration (or activation) of glial activity in ApoE4 carriers might prevent AD pathogenesis. With current technologies, the hypothesis that ApoE4 suppresses glial immune reactivity before AD onset is difficult to validate in humans as postmortem material normally reflects late disease states. It would, therefore, be highly relevant to evaluate, for example, immunoproteasome levels, MHC class I expression and lipid levels in healthy ApoE4 carriers early in life, such as trough tissue obtained from normal-pressure hydrocephalus biopsies^86^. The exact pathway connecting cholesterol levels to interferon pathways, the immunoproteasome and MHC class I antigen presentation also needs more study. Processes such as demyelination or phagocytosis of neuronal debris could contribute to increasing levels of cholesterol in astrocytes in the AD brain. Our data indicate that cholesterol can increase IRF1 levels, which is a master regulator of MHC class I expression. Cholesterol might intersect with interferon signaling at the plasma membrane in lipid rafts^87,88^ or through interactions with the cGAS–STING pathway^89–91^. However, our study here clearly shows that ApoE4 affects glial immune function and that this effect is mediated by ApoE4-induced changes in glial lipid metabolism and storage.

## Limitations

In vitro growth and medium composition are major regulators of lipid metabolism; hence, it was striking to see that iPS cell-derived brain cells largely recapitulate in vivo lipotypes. Specifically, for our iAstrocytes, the astrocytes were differentiated in medium consisting of serum that contains protein and lipid factors that are not present in the brain parenchyma under healthy conditions but penetrate the brain when the blood–brain barrier is impaired (such as in aging or AD^92–94^). To limit possible confounding effects of serum in the medium, we removed serum from our iAstrocytes during terminal experiments. In addition, we were able to recapitulate major reactive phenotypes in mature astrocytes isolated from mouse brain that were isolated and cultured without any serum (Extended Data Fig. 8). Our study also highlights significant variability across ApoE4 effects depending on the parental line in which the mutation is introduced (lipidomics effects in Fig. 3; proteomic and transcriptomic differences in Fig. 4). The effect of ApoE4 genotype is likely affected by genetic context, such as haplotype, gender or ethnicity. The variation between parental lines might be biologically meaningful. However, more studies with a higher number of isogenic sets will be required to address these questions. For the interpretation of our data, here, we focused on lipid classes (CE and TG) and Reactome pathways that were changed consistently across all isogenic sets and, where possible (for example, transcriptomics data), we compared our findings to previously published isogenic lines. We found that interferon signaling, MHC class I antigen presentation and ER–phagosome pathways were downregulated in ApoE4 iAstrocytes from all these studies (Fig. 4), indicating a robust and likely genetic context-independent effect of ApoE4 on the suppression of these immune pathways.

## Conclusion

Overall, our data highlight the important role of lipid (particularly cholesterol) metabolism in AD. We created a new tool (the Neurolipid Atlas) as a resource of neurolipidomic datasets for different cell types, mutations, neurodegenerative diseases and model organisms. As a proof of concept, we show that iNeurons, iAstrocytes and iMicroglia have distinct lipidomes that recapitulate in vivo lipotypes. Our data solidify the link between AD and cholesterol, further establishing CE accumulation as a hallmark of AD. Lastly, we show that cholesterol regulates astrocytic immune function, which is impaired by the genetic AD risk variant ApoE4. We will continue to grow the Neurolipid Atlas database and invite other researchers to upload their data as well, working toward a detailed understanding of the role that lipids have in brain health and disease.

## Methods

All research complied with the relevant ethical regulations and institutional guidelines at the Vrije Universiteit.

### iPS cell culture

Isogenic Kolf2.1J (APOE3/3), Kolf2.1J C112R Hom3 (APOE4/4 set 1) and Kolf2.1J C112R Hom2 (APOE4/4 set 2) human iPS cells were a kind gift from iNDI (donor: 57-year-old man). Kolf2.1J C112R Hom2 (APOE4/4 set 2) corresponds to JIPSC001142 available through iNDI. Isogenic BIONi037-A (APOE3/3) and BIONi037-A4 (APOE4/4) human iPS cell lines were obtained through EBISC (donor: 77-year-old woman). iPS cells were cultured in six-well plates precoated with 120–180 µg ml^−1^ Geltrex (Fisher Scientific, A1413302) in Gibco Essential 8 medium (E8; Fisher Scientific, 15190617) + 0.1% penicillin and streptomycin (P/S; Fisher Scientific, 11548876), with daily full medium refreshments. iPS cell colonies grown until 90% confluency were dissociated using 1 mM EDTA (Invitrogen, 15575-038) in 1× PBS (VWR, 392-0434) and replated in E8 medium supplemented with 5 μM ROCK inhibitor (RI; Tebu-Bio, Y27632). The genomic integrity of iPS cell lines was periodically tested using single-nucleotide polymorphism (SNP) arrays, as described below. In addition, cell cultures were regularly tested for *Mycoplasma* contamination.

### QC of iPS cells

DNA from cell cultures was isolated using a ReliaPrep genomic DNA tissue miniprep system (Promega, A2052). Samples were processed by the Global Screening Array (GSA) Consortium Project at Erasmus MC Rotterdam on the Illumina GSA beadchip GSA MD version 1. SNP data were processed and annotated with Illumina GenomeStudio software (Illumina). The iPsychCNV package was used for copy-number variant (CNV) calling, which integrates B allele frequency distribution and log R ratio to reduce false-positive detection (10.5281/zenodo.46235)^95^. CNVs larger than 25 kB and containing more than 100 SNPs were flagged and compared to gene lists associated with brain development and synapse Gene Ontology terms. In addition, DNA from iAstrocytes in each experiment was isolated to confirm the APOE genotype.

### iPS cell differentiation to iNeurons

NGN2 transcription-based iPS cell differentiation to neurons was according to a previous study^44^. iPS cells were infected in suspension (in E8 + RI) with ultrahigh-titer lentiviral particles provided by ALSTEM, encoding pTet-O-Ngn2-puro (Addgene, 52047) and FUΔGW-rtTa (Addgene, 19780). To start neuronal induction, 100,000 infected iPS cells per cm^2^ were plated in N2-supplemented medium (DMEM/F12 + GlutaMAX (Thermo Fisher, 31331093), 3 g L^−1^ d-glucose (Thermo Fisher, A2494001), 1% N2 supplement B (StemCell Technologies, 07156) and 0.1% P/S) supplemented with 5 µM RI, 2 μg ml^−1^ doxycycline hyclate (Sigma-Aldrich, D9891) and dual SMAD inhibitors (100 nM LDN193189 (Stemgent, 04-0074), 10 μM SB431542 (Tebu-Bio, T1726) and 2 μM XAV939 (Sigma-Aldrich, X3004)). On day 2, 100% of the medium was refreshed (including all day 1 supplements except RI) and 3 µg ml^−1^ puromycin was added (Cayman Chemical, 13884-25). On day 3, 100% medium was exchanged for N2-supplemented medium with doxycycline hyclate, puromycin and 10 µM 5-fluoro-2′-deoxyuridine (Sigma-Aldrich, F0503). Six-well plates were coated with 20 µg ml^−1^ poly(l-ornithine) (PLO; Sigma-Aldrich, P3655) overnight at room temperature (RT), followed by three wash steps with PBS on day 4. PLO-coated wells were subsequently coated with 5 µg ml^−1^ laminin (lam; BioTechne, 3400-010-02) for 2–4 h at 37 °C. iNeurons were washed with 1× PBS before dissociation with Accutase (Merck, SCR005) for 5 min at 37 °C. iNeurons were collected in DMEM (VWR, 392-0415P) and pelleted by a 5-min spin at 180*g*. iNeurons were resuspended and plated at 600,000 cells per well in PLO–lam-coated six-well plates in Neurobasal medium (NBM; Fisher Scientific, 11570556), supplemented with 200 mM GlutaMAX (Thermo Fisher, 35050038), 3 g L^−1^ d-glucose (Thermo Fisher, A2494001), 0.5% nonessential amino acids (NEAA; Fisher Scientific, 11350912), 2% B27 (Fisher Scientific, 17504044), 0.1% P/S, 10 ng ml^−1^ brain-derived neurotrophic factor (StemCell Technologies, 17189321), 10 ng ml^−1^ ciliary neurotrophic factor (CNTF; Peprotech, 450-13) and 10 ng ml^−1^ glial-cell-derived neurotrophic factor (StemCell Technologies, 78058.3). iNeurons were cultured at 37 °C and 5% CO_2_ and medium was replaced with 50% fresh medium once a week.

The purity of iNeuronal cultures was assessed by determining the percentage of MAP2-positive cells in culture. Columbus version 2.5.2 (PerkinElmer) was used to detect intact nuclei and quantify MAP2 signal intensity in a 6-μm ring around the nucleus. The MAP2-positive neuron population was determined as the percentage of MAP2-positive cells with a signal-to-noise ratio (SNR) > 3. The SNR indicates the s.d. of the signal above the mean background signal and was calculated by subtracting the mean of the background from the MAP2 signal in the ring region around the nucleus and dividing the result by the s.d. of the mean background signal.

### iPS cell differentiation to iAstrocytes

iPS cells were differentiated to neuronal progenitor cells (NPCs) according to a previous study^96^. On day 1, iPS cells were plated at 100% density in six-well plates in NMM medium (50% DMEM/F12 + GlutaMAX (Thermo Fisher, 31331093), 50% NBM, 100 mM GlutaMAX, 0.5% N2 supplement B, 1% B27, 0.5% ITS-A (Thermo Scientific, 51300044), 0.5% NEAA, 0.08% 2-mercaptoethanol (Fisher Scientific, 11528926) and 1% P/S supplemented with 10 µM SB431542 and 0.5 µM LDN193189. Complete medium was replaced daily for 7 days. On day 8, cells were expanded to PLO–lam-coated 6-cm dishes. Then, 1 ml of EDTA per well was added after one PBS wash and cells were incubated at 37 °C for 3–4 min. Cells were collected in clumps using a cell scraper and plated in 5 ml of complete NMM medium supplemented with 5 μM RI. On day 9, medium was exchanged for plain NMM medium without inhibitors after one PBS wash. This medium was refreshed daily for two more days. On day 12, the medium was exchanged for NMM medium supplemented with 10 ng ml^−1^ fibroblast growth factor (FGF; Peprotech, 100-18B). This medium was refreshed daily for two more days. On day 15, cells were incubated in Accutase after one PBS wash for 5 min at 37 °C and collected in NMM + 5 µM RI. After a 5-min spin at 1,000 rpm, the pellet was resuspended in NMM supplemented with FGF and RI before plating the NPC cells (passage 1, P1) in two PLO–lam-coated 10-cm dishes. The medium was refreshed daily with NMM + FGF for the next 3 days. NPCs were maintained at high density and refreshed every 2–3 days. NPCs were plated for control stainings (Nestin/Pax6) at P4 to confirm NPC identity, after which astrocyte differentiation was started according to Fong et al.^45^. One confluent 10-cm dish of NPCs was washed with 1× PBS before adding 9 ml of NMM + FGF. Cells were collected in clumps by cell scraper and transferred at 3 ml per well to a noncoated six-well plate. Plates were placed on an orbital shaker (90 rpm) in a 37 °C incubator. After 24 h, when tiny neurospheres had formed, 5 µM RI was added per well. Then, 48 h later, the medium was changed back to NMM without FGF. Next, 1 week after cell scraping of the NPCs, the NMM medium was exchanged for astrocyte medium (AM; ScienCell, 1801); subsequently, the medium was refreshed three times a week for the next 2 weeks. Neurospheres from three wells were collected and plated in one PLO–lam-coated 10-cm dish. iAstrocytes differentiated from the neurospheres were passaged to uncoated 10-cm dishes using Accutase and maintained in AM + 2% FBS (ScienCell, 1801/0010) until P4. iAstrocytes were plated for experiments when they were between P4 and P12. Columbus version 2.5.2 (PerkinElmer) was used to detect intact nuclei and quantify AQP4 and vimentin intensity in each cell. The purity of the astrocyte population was determined as the percentage of cells with an SNR > 3 over the secondary antibody control. The SNR indicates the s.d. of the signal above the secondary antibody control and was calculated by subtracting the mean of the secondary antibody signal from the AQP4 or vimentin mean intensity per cell and dividing the result by the s.d. of the mean secondary antibody signal.

### iPS cell differentiation to iMicroglia

iMic were generated according to a previous study^39^, with small modifications. In brief, iPS cells were detached with Accutase (Gibco) and collected as a single-cell suspension. After centrifugation (5 min, 300*g*, RT), 2.5 million cells were plated into 24-well AggreWell800 plates (StemCell Technologies; pretreated with anti-adherence rinsing solution) in 2 ml of embryoid body (EB) induction medium (mTeSR^+^ (StemCell Technologies) + 20 ng ml^−1^ stem cell factor (R&D Systems) + 50 ng ml^−1^ bone morphogenetic protein 4 (BMP4; Miltenyi) + 50 ng ml^−1^ vascular endothelial growth factor (Miltenyi), supplemented with 10 μM Y27632 (StemCell Technologies) for the first 24 h) per well to generate EBs. To allow the formation of EBs, cells remained in AggreWell plates with daily 75% medium changes for 5 days. After 5 days, EBs were isolated and equally distributed to two six-well plates (Corning) in 2 ml of EB differentiation medium (X-Vivo 15 (Lonza), 2 mM GlutaMAX (Gibco), 0.55 mM β-mercaptoethanol (Gibco), 100 U per ml and 100 μg ml^−1^ P/S (Thermo Fisher Scientific), 25 ng ml^−1^ IL-3 (Miltenyi Biotec) and 100 ng ml^−1^ macrophage colony-stimulating factor (Miltenyi Biotec)) per well. The EBs were kept in EB differentiation medium at 37 °C and 5 % CO_2_ with full medium changes every 7 days. After 2–3 weeks, nonadherent microglial precursor cells (pre-iMics) started to be released into the medium from EBs. Pre-iMics were isolated during regular medium changes by collecting the supernatant medium and straining through a 40-µm cell strainer (Greiner). Pre-iMics isolated in weeks 3–6 after emergence were pooled and sustained in EB differentiation medium in T75 flasks (Corning) with weekly medium changes. Once sufficient cell numbers were collected, pre-iMics were plated at 15,000 cells per cm^2^ in T175 flasks (Sarstedt) in iMic medium (50% advanced NBM (Gibco), 50% advanced DMEM/F12 (Gibco), 1× B27 supplement with vitamin A (Gibco), 2 mM GlutaMAX (Gibco), 0.1 mM β-mercaptoethanol (Gibco), 100 ng ml^−1^ IL-34 (Miltenyi Biotec) and 20 ng ml^−1^ macrophage colony-stimulating factor (Miltenyi Biotec)) and differentiated to iMics for 14 days. For each line, four replicates were plated and processed in parallel. iMics were cultivated at 37 °C and 5% CO_2_ with three full medium changes per week. On day 14 (or on days 0, 3 or 7 for qPCR), iMics were washed briefly with PBS and detached with Accutase for 6–7 min at 37 °C until cells detached upon tapping the flask. Cells were collected with wash buffer (advanced DMEM/F12 (Gibco) + 0.1% BSA fraction V (Gibco)) and centrifuged at 300*g* (5 min, RT) before they were resuspended in PBS and counted using a hemocytometer (Neubauer Zählkammer Improved, Bard). Appropriate volumes containing 1 million cells were transferred to 1.5-ml microcentrifugation tubes (Eppendorf) and centrifuged at 400*g* (4 °C, 5 min). The supernatant was aspirated and cell pellets were frozen to −80 °C before shipment for lipidomics analysis.

The quality and purity of EB cultures were assessed by flow cytometry. Pre-iMicroglias were isolated as described above from EB cultures. Cells were centrifuged at 300*g* (5 min, RT) and resuspended in 100 µl of fluorescence-activated cell sorting (FACS) buffer (PBS, 0.1% BSA fraction V (Gibco) and transferred into low-protein-binding 1.5-ml Eppendorf tubes. Cells were incubated with human Fc block (BD Biosciences, 564219) for 15 min at 4 °C in the dark on a rotor before 2 µl of each FACS antibody was added: APC anti-human CD45 antibody, mouse IgG1, HI30 (BioLegend, 304011) and Brilliant violet 421 anti-mouse/human CD11b antibody, clone M1/70, rat IgG2b (Biolegend, 101235). Pre-iMics were incubated with antibodies for 30 min at 4 °C in the dark on a rotor. Next, 1 ml of FACS buffer was added for washing and cells were centrifuged for 5 min (300*g*, 4 °C). The supernatant was removed and the cell pellet was resuspended in 500 µl of FACS buffer. Cells were analyzed using a Sony SH800S cell sorter (Sony Biotechnologies). In total, 20,000 cells per sample were analyzed. Unstained cells served as a gating control. Data analysis was conducted in FlowJo (BD Biosciences).

### Mouse astrocytes

All animals were bred and housed according to institutional and Dutch governmental guidelines and regulations. Mouse astrocytes were isolated and cultured according to a protocol adopted from Clayton et al.^97^. Timed pregnant C57bl6j mice were purchased from Charles River or bred in house. Brains were extracted from both male and female pups (E18–P3). Cortices were isolated after the removal of meninges; cortices from each brain were digested in Papain (LK003150, Worthington) at 37 °C for 30–45 min and triturated with supplemented DMEM + DNAse. The cell suspension was centrifuged at 300*g* for 5 min and cell pellet was resuspended in 50% DMEM/F12 (Gibco, 31331093) and 50% NBM (Gibco, 11570556) supplemented with 1% N2 supplement (StemCell Technologies, 07156), 2% B27 (Fisher Scientific, 17504044), 1% GlutaMAX (Life Technologies, 35050038), 1% NEAA (Fisher Emergo, 11350912), 1% P/S (Fisher Emergo, 11548876), 0.3% glucose (Life Technologies), 5 μg ml^−1^ *N*-acetyl cysteine (Sigma, A9165), 20 ng ml^−1^ FGF2 (Peprotech, 100-18B), 10 ng ml^−1^ CNTF (Peprotech, 450-13), 10 ng ml^−1^ BMP4 (Peprotech, 120-05ET) and 5 ng ml^−1^ heparin-binding epidermal growth factor (hbEGF; Peprotech, 100-47) and filtered through a 100-μm filter before plating cells in a 10-cm dish (one brain per dish) that was precoated for 24 h with PLO (Sigma, P3655) followed by lam (BioTechne, 3400-010-02) for 2 h at 37 °C. On day 2, plates were washed with 1× PBS and the medium was replaced by expansion medium, 50% DMEM/F12 (Gibco, 31331093) and 50% NBM (Gibco, 11570556) supplemented with 1% N2 supplement (StemCell Technologies, 07156), 1% GlutaMAX (Life Technologies, 35050038), 1% NEAA (Fisher Emergo, 11350912), 1% P/S (Fisher Emergo, 11548876), 0.3% glucose (Life Technologies), 5 μg ml^−1^ *N*-acetyl cysteine (Sigma, A9165), 20 ng ml^−1^ FGF2 (Peprotech, 100-18B), 10 ng ml^−1^ CNTF (Peprotech, 450-13), 10 ng ml^−1^ BMP4 (Peprotech, 120-05ET) and 5 ng ml^−1^ hbEGF (Peprotech, 100-47). The medium was replaced every 2 or 3 days until plates were confluent (9–14 days). Cells were lifted by TrypLE and collected in DMEM/F12 (Gibco, 31331093) before spinning at 300*g* for 5 min. Pellets were resuspended and cells were replated for an experiment in 50% DMEM/F12 (Gibco, 31331093) and 50% NBM (Gibco, 11570556) supplemented with 1% N2 supplement (StemCell Technologies, 07156), 1% GlutaMAX (Life Technologies, 35050038), 1% NEAA (Fisher Emergo, 11350912), 1% P/S (Fisher Emergo, 11548876), 0.3% glucose (Life Technologies), 5 μg ml^−1^ *N*-acetyl cysteine (Sigma, A9165), 20 ng ml^−1^ FGF2 (Peprotech, 100-18B), 10 ng ml^−1^ CNTF (Peprotech, 450-13), 10 ng ml^−1^ BMP4 (Peprotech, 120-05ET) and 5 ng ml^−1^ hbEGF (Peprotech, 100-47); alternatively, cells were frozen in CryoStor (StemCell Technologies, 07959).

For reactive astrocyte analysis mouse astrocytes were plated. Then, 24 h after plating, the medium was replaced by experiment medium, 50% DMEM/F12 (Gibco, 31331093) and 50% NBM (Gibco, 11570556) supplemented with 1% N2 supplement (StemCell Technologies, 07156), 1% GlutaMAX (Life Technologies, 35050038), 1% NEAA (Fisher Emergo, 11350912), 1% P/S (Fisher Emergo, 11548876), 0.3% glucose (Life Technologies), 5 μg ml^−1^ *N*-acetyl cysteine (Sigma, A9165) and 5 ng ml^−1^ hbEGF (Peprotech, 100-47). This condition constituted the no-FBS condition (−FBS). In parallel, astrocytes were cultured identically but 2% FBS was added to the culture medium (+FBS condition, to mimic the human astrocyte growth condition). Cells were cultured in their respective −FBS or + FBS medium for 5 days. After 5 days, the culture medium (+FBS or −FBS) was replaced by experiment medium without FBS for 24 h. After these 24 h, the medium was again replaced with experiment medium (without FBS) with or without the addition of the reactive cocktail (30 ng ml^−1^ TNF (300-01A, Peprotech), 3 ng ml^−1^ IL-1α (AF-200-01A, Peprotech) and 400 ng ml^−1^ C1q (204876, Sigma-Aldrich)). After 24 h, cells were lifted by TrypLE and pellets were collected and snap-frozen for lipidomics analysis. Each lipidomics or qPCR datapoint represents astrocytes from one independent mouse brain.

### Postmortem brain sample lipidomics

Lipidomic analysis was undertaken on human postmortem brain material including frontal cortex gray matter, frontal cortex white matter and cerebellum tissue from 13 control donors and 20 donors with AD. Brain tissue was obtained from the Queen Square Brain Bank, University College London Queen Square Institute of Neurology. All donor information, including postmortem delay, age, sex, APOE genotype and pathological information, is listed in Supplementary Table 2. Ethical approval for the study was obtained from the National Health Service research ethics committee in accordance with the human tissue authority’s code of practice and standards (license number 12198). Processing of postmortem samples for lipidomics was carried out as follows. After adding stainless-steel beads and liquid chromatography–mass spectrometry (LC–MS)-grade water, brain samples were homogenized using a Next Advance bullet blender. From these homogenized samples, aliquots containing the equivalent of 5 mg of tissue were prepared as described below.

### Lipidomic analysis

Lipidomics analysis followed standardized, quantitative protocols^46,98^. Briefly, 25 µl of Lipidyzer internal standard mix containing 54 deuterated standards was added to the cell pellet or 5-mg homogenized sample and extraction followed a methyl *tert*-butyl ether-based protocol. After drying under a gentle stream of nitrogen, samples were dissolved in running buffer (methanol and dichloromethane 1:1, containing 10 mM ammonium acetate) and injected into the Lipidyzer platform, consisting of a Sciex QTrap 5500 MS instrument equipped with a SelexION DMS interface and Nexera X2 ultrahigh-performance LC system. The order of samples was randomized before each batch was run on the Lipidyzer platform. SLA software was used to process data files and report the lipid class and species concentration and composition values^47^. Lipidyzer data analysis was further accomplished using SODA-light as a built-in data browser for the Neurolipid Atlas repository. Lipid species concentration datasets were imported and filtered, with individual species required to have a minimal intensity of two times the blank in at least 80% of all samples measured. If lipid species were absent or below two times the blank in >20% of all samples, they were removed. An exception was made for lipid species that were uniquely present in one group; if a lipid species was present in at least 60% of the samples (with a minimal intensity of two times the blank) within one of the experimental groups, the lipid species was reintroduced for the analysis. Because a dataset can have several grouping variables (for example, genotype, treatment and sample type), a new group variable is created by concatenating all grouping variables. This new group variable was used as the group variable for the blank filtering. No missing value imputation was applied. The SLA control software, including all up-to-date dictionaries and isotope correction algorithms can be found on Github (https://github.com/syjgino/SLA). SODA-light is a development branch of iSODA^99^ (https://github.com/ndcn/soda-ndcn) and part of the Neurolipid Atlas.

### Neurolipid Atlas coding and code availability

SODA-light was forked as a lipidomics-only instance of iSODA^99^, a multiomics data visualization and integration application developed on R 4.4.0. As such, SODA-light is designed for efficient data exploration, providing interactive plots with extensive flexibility in terms of input data, analytical processes and visual customization. The code for SODA-light is available on GitHub (https://github.com/CPM-Metabolomics-Lipidomics/soda-light). SODA-light version 0.2 was used for the generation of all figures in this manuscript.

### Adding data to the Neurolipid Atlas and metadata formatting

Lipidyzer data analyzed by SLA can be added to the Neurolipid Atlas website by contacting the corresponding authors or the email address at https://neurolipidatlas.com/. Upload requires concentrations of individual lipid species in a sample of interest, along with blank (empty) samples and QCs (for example, serum). Human data need to be anonymized. Metadata need to be provided in the format shown in Supplementary Table 3. Metadata items in bold (left column) need to be completed by the lab that provides the data. For adding other types of quantitative (targeted) lipidomics data, please contact the corresponding authors.

### Phospholipid, CE and TG saturation analysis

To investigate differences in saturation of lipid classes between groups, the sum of the concentration of the lipid species with identical numbers of double bonds within the TGs, CEs or within all phospholipid classes was calculated. These summed values were normalized over total lipid concentration. Afterward, the fold change from each sample was calculated over the mean of the control samples.

### Lipidomics of iAstrocytes

For lipidomics, fully differentiated iAstrocytes were plated on day 1 at 17,000 cells per cm^2^ in AM + 2% FBS in uncoated 10-cm dishes. On day 2, the medium was replaced by AM without FBS after one PBS wash. After 24 h, on day 3, iAstrocytes were collected by Accutase dissociation after one PBS wash and counted. Cells were pelleted at 500,000 per vial using a centrifuge with a swing-out rotor. For reactive iAstrocyte lipidomics, the setup was identical; however, on day 3, the medium was replaced by AM without FBS supplemented with a reactive cytokine cocktail (30 ng ml^−1^ TNF (300-01A, Peprotech), 3 ng ml^−1^ IL-1α (AF-200-01A, Peprotech) and 400 ng ml^−1^ C1q (204876, Sigma-Aldrich)) or AM without FBS supplemented with an equal amount of PBS + 0.1% BSA (Tebu-Bio, 1501) as a control. On day 4, iAstrocytes were collected by Accutase dissociation after one PBS wash and counted. Cells were pelleted at 500,000 per vial using a centrifuge with a swing-out rotor. For one experiment comparing ApoE4 to ApoE3 iAstrocytes and one experiment comparing reactive to control iAstrocytes, lipidomics was performed as part of a multiomics experiment, as discussed below. Each lipidomics experiment included two or three replicate wells of iAstrocytes per condition.

### Experimental setup for iAstrocyte multiomics: ApoE4 versus ApoE3

For integrative analysis, we generated samples for lipidomics and proteomics from the same preparation of BIONi037 (lipidomics experiment BIONi037 replicate I) and Kolf2.1J (lipidomics experiment Kolf2.1J set 1, replicate II) isogenic iAstrocytes. Transcriptomics was performed on a separate preparation for both isogenic sets (described below). To generate lipidomics and proteomics from one preparation, on day 1, fully differentiated iAstrocytes were plated at 17,000 cells per cm^2^ in AM + 2% FBS in uncoated 10-cm dishes. On day 2, the medium was replaced by AM without FBS after one PBS wash. After 24 h, on day 3, iAstrocytes were collected by Accutase dissociation after one PBS wash and counted. iAstrocytes were pelleted at 500,000 per vial separately for lipidomics and proteomics. Three replicate samples were generated for lipidomics and four replicate samples were generated for proteomics. iAstrocyte pellets were stored at −80 °C until shipment for further analysis. Generation of transcriptomic samples was performed under identical conditions (1,000,000 cells per vial; three replicates) for the Kolf2.1J isogenic set (set 1), whereas, for the BIONi037 isogenic set, the cells were kept in AM medium + 2% FBS.

### Experimental setup for iAstrocyte multiomics: reactive versus control

Similarly to above, lipidomic and proteomic samples were generated from the same preparation of reactive versus control iAstocytes for integrative analysis. Day 1 (fully differentiated) BIONi037-A (ApoE3/3) or Kolf2.1J set 1 (ApoE3/3) astrocytes were plated at 17,000 cells per cm^2^ in AM + 2% FBS in uncoated 10-cm dishes. On day 2, the medium was replaced by AM without FBS after one PBS wash. On day 3, the medium was replaced by AM without FBS supplemented with a reactive cytokine cocktail (30 ng ml^−1^ TNF (300-01A, Peprotech), 3 ng ml^−1^ IL-1α (AF-200-01A, Peprotech) and 400 ng ml^−1^ C1q (204876, Sigma-Aldrich)) or AM without FBS supplemented with an equal amount of PBS + 0.1% BSA (Tebu-Bio, 1501) as a control. After 24 h, iAstrocytes were collected by Accutase dissociation after one PBS wash. iAstrocytes from each replicate dish were divided over two vials in which (1 million for Kolf2.1J or 500,000 for BIONi037-A) iAstrocytes were pelleted for proteomic and lipidomic analysis. Cell pellets were stored at −80 °C before further processing.

For iAstrocytes on the WTC11 background, differentiation was performed as previously described^100^, with minor modifications. Briefly, WTC11 iPS cells were edited to introduce a doxycycline-induced cassette driving proastrocyte transcription factors NFIA and SOX9. These iPS cells were differentiated into NPCs using dual SMAD inhibition and EB formation. NPCs were purified by FACS for CD133^+^CD271^−^ populations. Purified NPCs were further differentiated into iAstrocytes by doxycycline treatment (2 μg ml^−1^; Millipore Sigma, D9891) and exposure to AM (ScienCell, 1801) for 20 days. For experiments using serum-containing growth conditions, day 20 iAstrocytes were plated at 20,000 cells per cm^2^ in phenol-red-free AM (prfAM) (ScienCell, 1801-prf) overnight, with a full medium change to fresh prfAM on day 21. The medium was changed to fresh prfAM every 2 days. On day 25, cells were treated with 30 ng ml^−1^ TNF (300-01A, Peprotech), 3 ng ml^−1^ IL-1α (AF-200-01A, Peprotech) and 400 ng ml^−1^ C1q (204876, Sigma-Aldrich). After 24 h, the medium was removed, cells were washed with 1× Dulbecco’s (DPBS) and cell pellets were collected and stored at −80 °C before further processing for lipidomics. Studies with human iPS cells at the University of California, San Francisco were approved by the Human Gamete, Embryo and Stem Cell Research Committee. Informed consent was obtained from participants when the iPS cell lines were originally derived.

### Lipidomics of iPS cell-derived TMEM106B-knockout neurons

TMEM106B-knockout iPS cells, genetically engineered from the parental KOLF2.1J iPS cell line^59^, were obtained from iNDI^60^ through the Jackson Laboratory. These iPS cells, along with wild-type parental KOLF2.1J iPS cells, were cultured in feeder-free conditions on Matrigel in E8 medium (Life Technologies) and passaged by Accutase dissociation followed by E8 plus Chroman-I RI. A piggybac-based tet-on NGN2 transgene cassette was stably integrated into the genome of iPS cells as described previously (https://doi.org/10.17504/protocols.io.q26g744b1gwz/v1)^101^, followed by puromycin selection to eliminate iPS cells that did not successfully integrate the transgene. iNeurons were differentiated on six-well PLO-coated dishes, with a plating density of 500,000 cells per well on day 4, by doxycycline-induced expression of the NGN2 transgene, as described previously (https://doi.org/10.17504/protocols.io.8epv5969ng1b/v1)^102^. Cells were harvested on day 21 after doxycycline addition and snap-frozen before lipidomic analysis. Cells from two wells were combined into one 1.5-ml tube as one sample pellet. Experiments were conducted as three independent replicates with three samples per replicate.

### Lipidomics of control versus cholesterol-treated iAstrocytes

Day 1 (fully differentiated) BIONi037-A (ApoE3/3) astrocytes were plated at 26,000 cells per cm^2^ in AM + 2% FBS in uncoated six-well plates. On day 2, the medium was replaced by AM without FBS after one PBS wash. On day 3, the medium was replaced by AM without FBS supplemented with 50 µM methyl-β-cyclodextrin (MBCD)-coupled (water-soluble) cholesterol (C4951, Sigma-Aldrich) or AM without FBS supplemented with an equal amount of sterile water (TKF7114, Baxter) as a control. After 24 h, iAstrocytes were collected by Accutase dissociation after one PBS wash. iAstrocytes from each replicate well were pelleted for lipidomic analysis. Each lipidomics experiment included one or two replicate wells of iAstrocytes per condition. Cell pellets were stored at −80 °C before further processing.

### Sample preparation for proteomics analysis

Frozen pellets corresponding to ~500,000 cells were dissolved in 25 μl of PBS supplemented with one tablet of cOmplete Mini EDTA-free protease inhibitor per 50 ml. One volume equivalent of 2× lysis buffer (100 mM HEPES pH 8.0, 50 mM DTT and 4% (w/v) SDS) was added. Samples were sonicated in a Bioruptor Plus (Diagenode) for ten cycles with 1 min on and 30 s off with high intensity at 20 °C. Samples were heated for 5 min at 95 °C and a second sonication cycle was performed as described above. Samples were alkylated using freshly made 15 mM iodoacetamide (Sigma-Aldrich, I1149) for 30 min at RT in the dark. Subsequently, proteins were acetone precipitated and digested using LysC (PTMScan; Cell Signaling, 39003) and trypsin (Promega sequencing-grade, V5111), as described previously^103^. The digested proteins were then acidified with 10% (v/v) trifluoracetic acid and desalted using a Waters Oasis HLB µElution plate (30 µm; Waters, 186001828BA) following the manufacturer’s instructions. The eluates were dried down using a vacuum concentrator and reconstituted in 5% (v/v) acetonitrile and 0.1% (v/v) formic acid. Samples were transferred to an MS vial, diluted to a concentration of 1 µg µl^−1^ and spiked with iRT kit peptides (Biognosys, Ki-3002-2) before analysis by LC–MS/MS.

### Proteomics data acquisition

Peptides were separated in trap/elute mode using the nanoAcquity MClass ultrahigh-performance LC system (Waters) equipped with trapping (nanoAcquity Symmetry C18, 5 μm, 180 μm × 20 mm) and analytical (nanoAcquity BEH C18, 1.7 μm, 75 μm × 250 mm) columns. Solvent A was water and 0.1% formic acid while solvent B was acetonitrile and 0.1% formic acid. First, 1 μl of the samples (∼1 μg on column) were loaded with a constant flow of solvent A at 5 μl min^−1^ onto the trapping column. The trapping time was 6 min. Peptides were eluted through the analytical column with a constant flow of 0.3 μl min^−1^. During the elution, the percentage of solvent B increased nonlinearly from 0% to 40% in 120 min. The total run time was 145 min, including equilibration and conditioning. The LC instrument was coupled to an Orbitrap Exploris 480 (Thermo Fisher Scientific) using the Proxeon nanospray source. The peptides were introduced into the MS instrument using a Pico-Tip Emitter (outer diameter, 360 μm; inner diameter, 20 μm inner diameter; 10-μm tip; New Objective) heated at 300 °C and a spray voltage of 2.2 kV was applied. The capillary temperature was set at 300 °C. The radiofrequency ion funnel was set to 30%. For data-independent acquisition (DIA), full-scan MS spectra with a mass range of 350–1,650 *m*/*z* were acquired in profile mode in the Orbitrap with the resolution of 120,000 full width at half-maximum (FWHM). The default charge state was set to 3+. The filling time was set at a maximum of 60 ms with a limitation of 3 × 10^6^ ions. DIA scans were acquired with 40 mass window segments of differing widths across the MS1 mass range. Higher-energy collisional dissociation fragmentation (stepped normalized collision energy: 25%, 27.5% and 30%) was applied and MS/MS spectra were acquired with a resolution of 30,000 FWHM at a fixed first mass of 200 *m*/*z* after the accumulation of 3 × 10^6^ ions or after a filling time of 35 ms (whichever occurred first). Data were acquired in profile mode. For data acquisition and processing of the raw data, Xcalibur 4.3 (Thermo Fisher Scientific) and Tune version 2.0 were used.

### Proteomics data analysis

DIA raw data were analyzed using the directDIA pipeline in Spectronaut version 18 (Biognosys) with default settings besides the following parameters: protein LFQ method, QUANT 2.0; proteotypicity filter, only protein group specific; major group quantity, median peptide quantity; minor group quantity, median precursor quantity; data filtering, *Q* value; normalizing strategy, local normalization. The data were searched against UniProt (*Homo sapiens*, 20,375 entries) and a contaminants database (247 entries). The identifications were filtered to satisfy a false discovery rate (FDR) of 1% on the peptide and protein level. Relative protein quantification was performed in Spectronaut using a pairwise *t*-test performed at the precursor level followed by multiple-testing correction according to a previous study^104^.

### RNA-seq analysis

RNA isolation, QC, preprocessing and data analysis were performed as previously described from frozen pellets^105^. Briefly, total RNA was isolated from each sample using the Qiagen RNeasy mini kit. RNA samples for each participant were entered into an electronic tracking system and processed at the University of California, Irvine Genomics Research and Technology Hub. RNA was QCed using an Agilent Bioanalyzer and quantified by Nanodrop. RNA quality was measured as the RNA integrity number (RIN) and 260/280 and 260/230 ratios to evaluate any potential contamination. Only samples with RIN > 8 were used for library prep and sequencing. Library prep processing was initiated with total RNA of 1 µg using a Ribo-Zero gold ribosomal RNA depletion and Truseq Stranded total RNA kit. RNA was chemically fragmented and subjected to reverse transcription, end repair, phosphorylation, poly(A) tailing, ligation of barcoded sequencing adaptors and enrichment of adaptor-ligated complementary DNA (cDNA). RNA-seq libraries were titrated by qPCR (Kapa), normalized according to size (Agilent Bioanalyzer 2100 high-sensitivity chip). Each cDNA library was then subjected to Illumina (Novaseq 6000) paired-end, 100-cycle sequencing to obtain approximately 50–65 million paired-end reads. FASTQ files were subjected to QC and reads with quality scores > Q15 were collected. Raw reads were mapped to the GRCh38 reference genome using Hisat2 (version 2.2.1), QC, normalization and transformation before further exploratory and differential expression analysis. Raw counts were normalized and transformed using the ‘regularized log’ transformation pipeline from the R package DESeq2. Statistical analyses was performed in R and differentially expressed genes detected for each covariate using FDR or Bonferroni adjustment for multiple-testing correction. PCA was performed using the plotPCA function in R with default settings. Following regularized log transformation in DESeq2, the top 500 highly variable genes were used as input for PCA and clustering of samples. DESeq2 was used to assess the statistical difference between the ApoE genotypes. Subsequently, we used the differentially expressed genes for each comparison to perform gene set enrichment analyses using Webgestalt^106^.

### RNA-seq data comparison

Expression data of iAstrocytes from Tcw et al.^10^ and Lin et al.^8^ were analyzed and downloaded using GEO2R^32^. Tcw et al. performed bulk RNA-seq on four isogenic sets of APOE3/3 and APOE4/4 iAstrocytes, as well as bulk RNA-seq on seven APOE3/3 and six APOE4/4 population iAstrocyte lines. Lin et al. performed bulk RNA-seq on one isogenic set of APOE3/3 and APOE4/4 iAstrocytes. From all five isogenic sets and the population model, we gained the differential gene expression data of APOE4/4 versus APOE3/3. We calculated the average log_2_ fold change in ApoE4 versus ApoE3 of the pathways that were the top ten upregulated and downregulated pathways in our APOE4/4 versus APOE3/3 (BIONi037) transcriptomics for each line or the population data. We compared the directionality in all the lines and further explored the gene expression of the pathways that had the same directionality for all the comparisons.

### Experimental setup: baseline experiments

On day 1, iAstrocytes were plated at 30,000–40,000 cells per cm^2^ in 6-well, 12-well and 96-well plates depending on the specific experiment in AM + 2% FBS (ScienCell). Then, 24 h after plating (day 2), the medium was replaced by AM without FBS after one PBS wash. On day 3, if needed, medium was collected and stored at −20 °C until further analysis by mesoscale discovery (MSD) cytokine ELISA. Attached iAstrocytes were either fixed by 3.7% formaldehyde (FA; Electron Microscopy Sciences, 15681) for 10–15 min at RT and stored at 4 °C in 1× PBS for immunofluorescence staining, collected by Accutase dissociation for flow cytometry, lysed in Laemmli sample buffer with DTT (LSB; made in house) for western blot, lysed with RLY plus TCEP (Meridian, BIO-52073; Thermo Scientific, 20491) for qPCR or lysed in radioimmunoprecipitation assay (RIPA) buffer (made in house) for BCA analysis.

### Experimental setup: drug treatment experiments

On day 1, iAstrocytes were plated at 30,000–40,000 cells per cm^2^ in 6-well, 12-well and 96-well plates depending on the specific experiment in AM + 2% FBS (ScienCell). Then, 24 h after plating (day 2), the medium was replaced by AM without FBS after one PBS wash. On day 3, iAstrocytes were treated with 50 µM MBCD-coupled (water-soluble) cholesterol (C4951, Sigma-Aldrich), 0.5 µM avasimibe (PZ0190, Sigma-Aldrich), the reactive cytokine cocktail (30 ng ml^−1^ TNF (300-01A, Peprotech), 3 ng ml^−1^ IL-1α (AF-200-01A, Peprotech) and 400 ng ml^−1^ C1q (204876, Sigma-Aldrich)) (dose 1) or lower titrated doses of the cocktail indicated by 0.5 (15 ng ml^−1^ TNF, 1.5 ng ml^−1^ IL-1α and 200 ng ml^−1^ C1Q), 0.25, 0.125 etc. Where indicated, iAstrocytes were preincubated for 1 h with 0.5 µM avasimibe (PZ0190, Sigma-Aldrich), 10 µM MBCD-coupled (water-soluble) cholesterol (C4951, Sigma-Aldrich) or 0.5 µM atorvastatin (HY-17379, MedChemExpress) before combined incubation with one of the previously mentioned treatments. Then, 24 h later, on day 4, if needed, the medium was collected and stored at −20 °C until further analysis by MSD cytokine ELISA. Attached iAstrocytes were fixed by 3.7% FA for 10–15 min at RT and stored at 4 °C in 1× PBS for immunofluorescence staining, collected by Accutase dissociation for flow cytometry, lysed in LSB for western blot, lysed with RLY plus TCEP for qPCR or lysed in RIPA buffer for BCA analysis.

### MSD cytokine measurements

The medium was thawed and cellular debris was removed by a 5-min spin at 2,000*g*. Cytokine levels were determined by MSD Il-6 V-plex (K151QXD-2, MSD) according to the manufacturer’s protocol. Medium samples were analyzed either undiluted or diluted 1:5 or 1:10 in diluent 2 when iAstrocytes were treated with the reactive cocktail. Raw cytokine values per well were normalized over nuclei number per well on the basis of fluorescence staining of the fixed iAstrocytes in the plate or over protein content per well determined by a Pierce BCA protein assay kit (Thermo Scientific, 23225). The Pierce BCA protein assay was performed in a microplate as described in the user guide provided by Thermo Scientific.

### ELISA

The medium was thawed and tenfold concentrated with centrifugal filters (3-kDa molecular weight cutoff; Millipore, UFC200324). ApoE levels in the medium were determined using the human ApoE ELISA kit (Invitrogen, EHAPOE) according to the manufacturer’s protocol. Raw values were normalized over protein content per well determined by a Pierce BCA protein assay kit (Thermo Scientific, 23225). The Pierce BCA protein assay was performed in a microplate as described in the user guide provided by Thermo Scientific.

### Cholesterol secretion in the medium

On day 1, iAstrocytes were plated at 30,000–40,000 cells per cm^2^ in six-well plates in AM + 2% FBS (ScienCell). Then, 24 h after plating (day 2), the medium was replaced by AM without FBS after one PBS wash. On day 3, iAstrocytes were treated with or without the reactive cytokine cocktail (30 ng ml^−1^ TNF (300-01A, Peprotech), 3 ng ml^−1^ IL-1α (AF-200-01A, Peprotech) and 400 ng ml^−1^ C1q (204876, Sigma-Aldrich)). The medium was collected after 24 h on day 4 and stored at −20 °C before shipment for analysis. Cells in each well were collected for lipidomics analysis. Secreted cholesterol levels in the medium samples were normalized to total lipid levels in the corresponding iAstrocyte cell pellets. For total cholesterol analysis, 1.0 ml of astrocyte conditioned medium was freeze-dried overnight. Subsequently, lipids were hydrolyzed using 200 µl of a 0.1 M ethanolic sodium hydroxide solution (80% ethanol) under nitrogen atmosphere and total cholesterol quantification using gas chromatography–MS followed published protocols^107^.

### Immunofluorescence staining and imaging

After fixation, cells were permeabilized with 0.5% Triton X-100 (Fisher Scientific, T/3751/08) for 5 min at RT and blocked in PBS with 0.1% Triton X-100 and 2% NGS (Fisher Scientific, 11540526) for 30 min at RT or normal donkey serum (Biozol Diagnostika, LIN-END9000) in the case of microglia staining. Next, the iAstrocytes were incubated with the primary antibodies in blocking solution for 2 h at RT or overnight at 4 °C. The following primary antibodies were used: anti-Plin2 (15294-1-AP, Proteintech; 1:250), anti-AQP4 (AQP-004, Alomone Labs; 1:500), anti-GFAP (173 004, Synaptic systems; 1:1000), anti-vimentin (sc-6260, Santa Cruz Biotechnology; 1:1,000), anti-MAP2 (ab5392, Abcam; 1:500), anti-Smi 312 (SMI-312P-050, Eurogentec; 1:500), anti-Iba1 (NB100-1028, Novus Biologicals; 1:250), anti-PU.1 (MA5-15064, Thermo Fisher Scientific; 1:200) and anti-HLA class I heavy chain (kind gift from S. Neefjes; 1:250). After three washes in 1× PBS, the iAstrocytes were incubated with Alexa Fluor secondary antibodies (Invitrogen; 1:1,000), combined with DAPI (Carl Roth, 6843.1) and optional Lipidspot 488 in Fig. 6b (70065, Bio Connect; 1:1,000) in blocking solution, for 1 h at RT. Secondary antibodies used for microglia stainings were donkey anti-Goat Alexa-488 (Jackson ImmunoResearch 705-545-147) and donkey anti-rabbit Alexa-568 (Invitrogen, A10042). The iAstrocytes were washed three times in 1× PBS and either left in PBS to be imaged on the CellInsight CX7 LED Pro HCS platform (Fisher Scientific) or mounted on coverslips with Mowiol 4-88 (Sigma-Aldrich, 475904) for confocal imaging on a Nikon Ti-Eclipse microscope (Amstelveen), equipped with a confocal scanner model A1R+, using a ×40 oil-immersion objective (numerical aperture, 1.3). Image analysis was conducted using Columbus version 2.5.2 (PerkinElmer) after imaging on the Cell Insight CX7 and using Fiji^108^ after confocal imaging on the Nikon Ti-Eclipse. Lipid droplet analysis was performed using Perkin Elmer Columbus image analysis software. Cells were first identified on the basis of segmentation of the nuclei and cytoplasm to select only those fully contained within the field of view. For quantification of lipid droplets, the ‘find spots’ function was applied for automated detection and counting of lipid droplets within each segmented cell. Alternatively, to assess antibody staining intensity, the ‘calculate intensity’ function was used to determine the mean fluorescence intensity per cell. The fluorescence intensity or lipid droplet number of at least 50 cells per well was quantified in each experiment.

### Flow cytometry of iAstrocytes

After Accutase dissocation, FACS buffer (DPBS + 2% FBS (Fisher Scientific, A5256701)) was added to a volume of 300 µl and the iAstrocytes were transferred to a round-bottom 96-well plate. After centrifuging for 1 min at 2,000 rpm, the iAstrocytes were stained with 1:50 phycoerythrin anti-human HLA-A, HLA-B and HLA-C antibody (BioLegend, 311406) for 30 min at 4 °C. Following another centrifugation step, the iAstrocytes were fixed with 2% FA (Sigma-Aldrich, P6148) for 15 min at RT. After a last centrifugation step, the iAstrocytes were transferred to FACS tubes and 10,000 events of each sample were analyzed using BD LSRFortessa X-20 (BD Biosciences). Data analysis was performed in FlowJo (BD Biosciences). First, a life gate was set using the forward and side scatter area, after which the single cells were gated using the forward scatter width and height (Supplementary Fig. 5). The geometric mean of the fluorescence intensity was used in the analyses.

### qPCR

RNA was isolated using the Isolate II RNA mini kit (BIO-52073, GC Biotech) according to the manufacturer’s protocol. RNA quantity and quality were analyzed by nanodrop. cDNA was generated by a SensiFAST cDNA synthesis kit (BIO-65054, GC Biotech) according to the manufacturer’s protocol. The SensiFast SYBR Lo-Rox kit was used for performing qPCR on a QuantStudio 3 real-time PCR system (Thermo Fisher Scientific) and analysis was performed according to the *ΔΔC*_*t*_ method to determine fold changes in RNA expression. For qPCR on iMic, the RNA Nucleospin Plus XS kit by Macherey-Nagel was used according to the kit’s instructions for cell lysis and RNA extraction. The Applied Biosystems cDNA reverse transcription kit (Thermo Fisher Scientific) was used according to the manufacturer’s protocol for reverse transcription. The list of primers used is provided in Supplementary Table 4.

### Western blot

After lysing the iAstrocytes with LSB, samples were denatured at 95 °C for 5 min. The samples were shortly vortexed and loaded onto 4–15% Criterion TGX stain-free gel (Bio-Rad, 5678085). After running the gel (90 V for 30 min followed by 150 V for 45 min), the gel was transferred to a low-fluorescence (LF) PVDF membrane using the Trans-Blot Turbo RTA Midi 0.45-µm LF PVDF transfer kit (Bio-Rad, 1704275). After blocking in 5% skim milk powder (Sigma-Aldrich, 115363) for 1 h at RT on a shaker, the membrane was incubated with antibodies to HLA class I heavy chain (kind gift from S. Neefjes; 1:250), TAP1 (kind gift from S. Neefjes; 1:500), TAP2 (kind gift from S. Neefjes; 1:3,000) and GAPDH (elabscience, E-AB-40337; 1:3,000) overnight at 4 °C on a rocking plate. The following day, the membrane was incubated with secondary antibodies IRDye 800CW or IRDye 680RD (LI-COR; 1:10,000) for 1 h at RT on a shaker. Afterward, the membrane was scanned using the LI-COR Odyssey Fc imaging system. Analysis was conducted using Image Studio Lite 5.2.5 software (LI-COR) by calculating the median intensity of the bands minus the background above and below the bands.

### Statistical analysis

Statistical analysis was performed in GraphPad Prism version 10.2.3 or RStudio version 2023.9.1. No statistical methods were used to predetermine sample sizes but our sample sizes are similar to those reported in previous publications. The statistical tests used and the number of biological replicates (separate astrocyte preparations) are annotated in the figures. In all graphs, open triangles or open circles indicate the mean result per experiment. Solid dots indicate the mean result of each replicate well in the experiments. The data met the assumptions of the statistical test used; normality and equal distribution were tested. Data collection and analysis were not performed blind to the conditions of the experiments because automated analysis was performed. Statistical analyses were performed on the mean values per experiment. For the comparison of lipid class changes in Figs. 2f, 3f and 5c and Extended Data Figs. 2g, 8i, 9e and 10e, statistical analyses were performed on the percentage of total lipid values for each group (not fold change). One ApoE4/4 sample from lipidomics iAstrocytes BIONi037 ApoE4 versus ApoE3 I was excluded as outlier. In Fig. 6j, one outlier experiment was removed on the basis of robust regression and outlier removal (*Q* = 1).

### Reporting summary

Further information on research design is available in the Nature Portfolio Reporting Summary linked to this article.

## Supplementary information

Supplementary Information Supplementary Figs. 1–5 and Tables 1–4.

Reporting Summary

## Source data

Source Data Fig. 4 Unprocessed western blots.

Source Data Extended Data Fig. 5 Unprocessed western blots.

## Data Availability

Bulk RNA-seq data of ApoE3/3 and ApoE4/4 iAstrocytes from the BIONi037-A and KOLF2.1J lines were deposited to the Gene Expression Omnibus under accession code GSE302826. All proteomics data were deposited to MassIVE: ApoE3/3 and ApoE4/4 iAstrocytes from the BIONi037-A line under accession code MSV000098665, ApoE3/3 and ApoE4/4 iAstrocytes from the KOLF2.1J line under accession code MSV000098666 and data of control and reactive iAstrocytes from the BIONi037-A and KOLF2.1J lines under accession code MSV000098668. All lipidomics data are available through https://neurolipidatlas.com/. Source data are provided with this paper.

## Code availability

The code for SODA-light is available from GitHub (https://github.com/CPM-Metabolomics-Lipidomics/soda-light).
