# Supplementary material for: The Neurolipid Atlas: a lipidomics resource for neurodegenerative diseases
Source: Nat Metab. 2025 Sep 22;7(10):2142–64. doi: 10.1038/s42255-025-01365-z (PMC12552125; doi:10.1038/s42255-025-01365-z)
Supplement: Supplementary file 1 — Supplementary Figs. 1–5 and Tables 1–4. [file 42255_2025_1365_MOESM1_ESM.pdf]

nature metabolism

Supplementary information https://doi.org/10.1038/s42255-025-01365-z

The Neurolipid Atlas: a lipidomics resource for neurodegenerative diseases

In the format provided by the authors and unedited

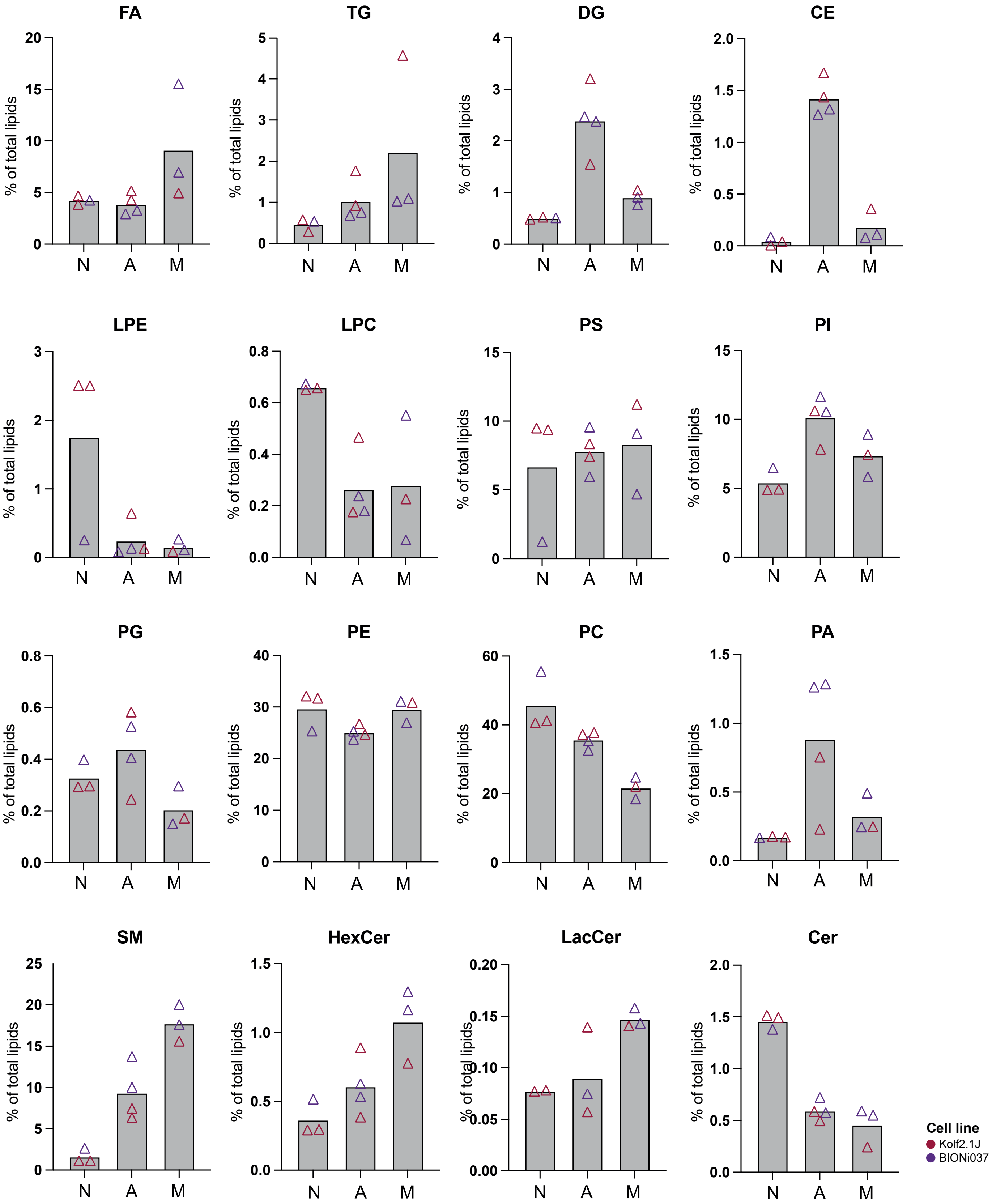

Supplementary Figure 1. Lipotypes of human iPSC-derived neurons, astrocytes and microglia (BIONi037). Each datapoint shows the abundance of the respective lipid class (as % of total lipids) from one independent experiment. N (Neurons) N=3, A (Astrocytes) N=4, M (Microglia) N=3. Data was derived from multiple (separate) lipidomics runs and not batch corrected to show the range in variation of samples and measurements.

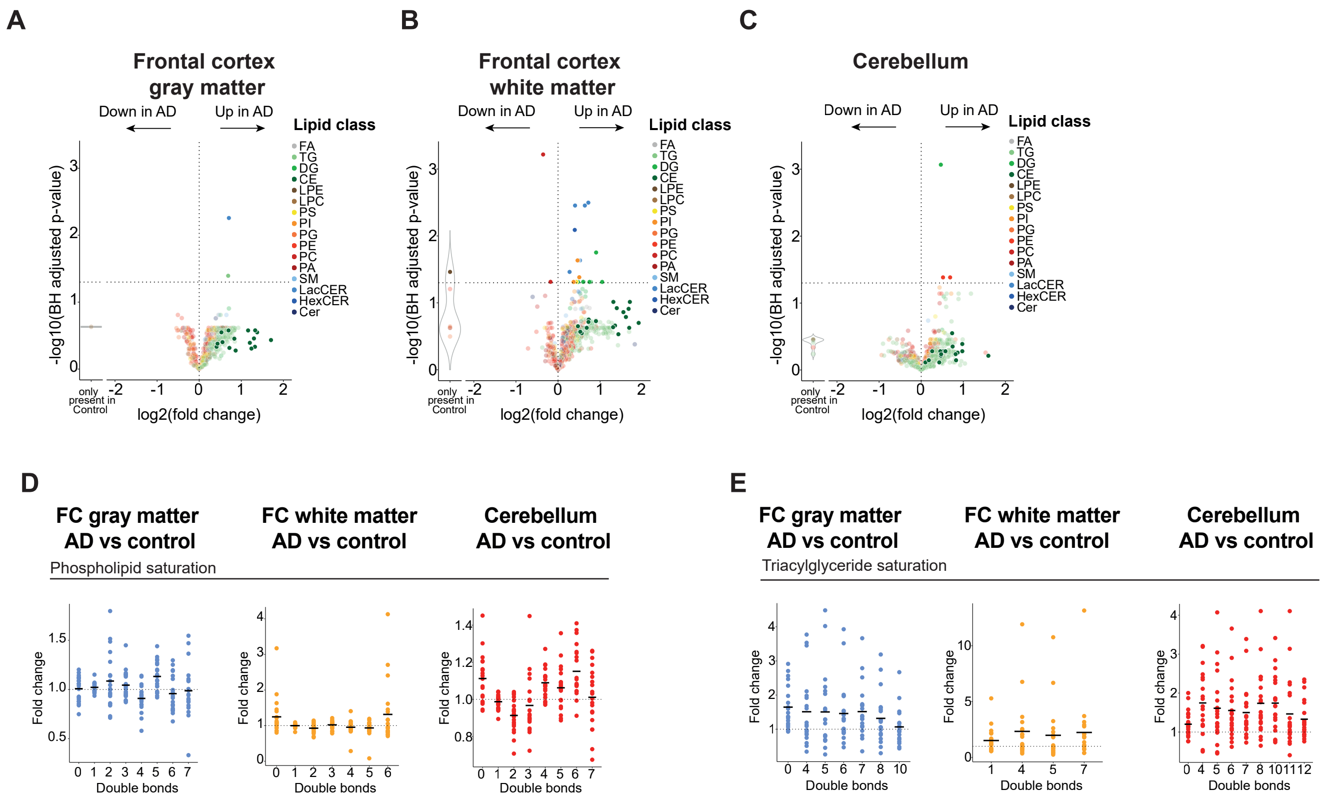

Supplementary Figure 2. Extended analysis of human (AD) brain lipidomics at the species level A-C) Volcano plots of individual lipid species in AD vs control brain tissue for FC gray matter (A), FC white matter (B) and cerebellum (C) as a percentage of the total lipidome. CE species are highlighted. D-E) Fold change of phospholipid (D) and TG (E) species with indicated number of double bonds in AD vs control samples from FC gray matter, FC white matter and cerebellum from % of total lipids. Mean.

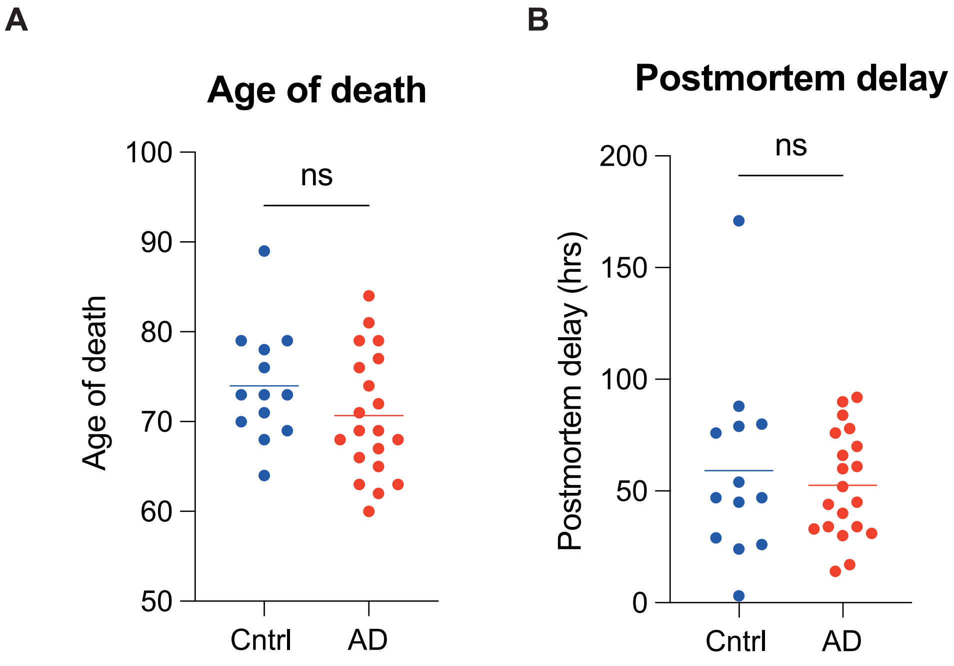

Supplementary Figure 3. Metadata human postmortem brain tissue. Age of death of each donor (A) and Postmortem delay for each donor tissue (B) are plotted. Mean. Two-sided unpaired t-test.

Bioni037A iAstrocytes

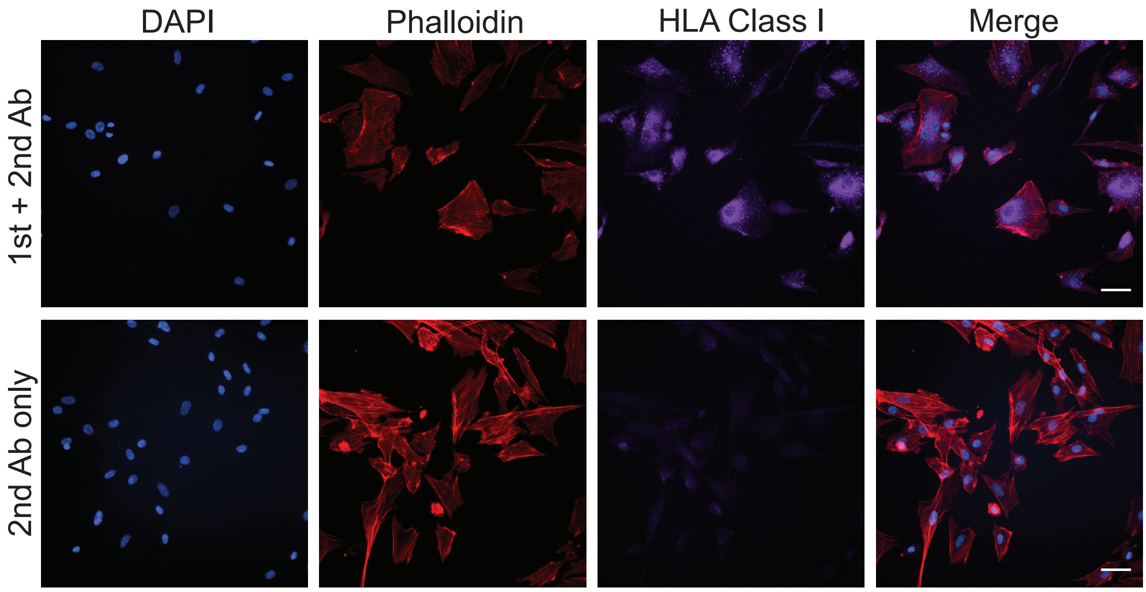

Supplementary Figure 4. 2nd antibody only control for HLA Class I antibody staining. Scale bar = 50μm.

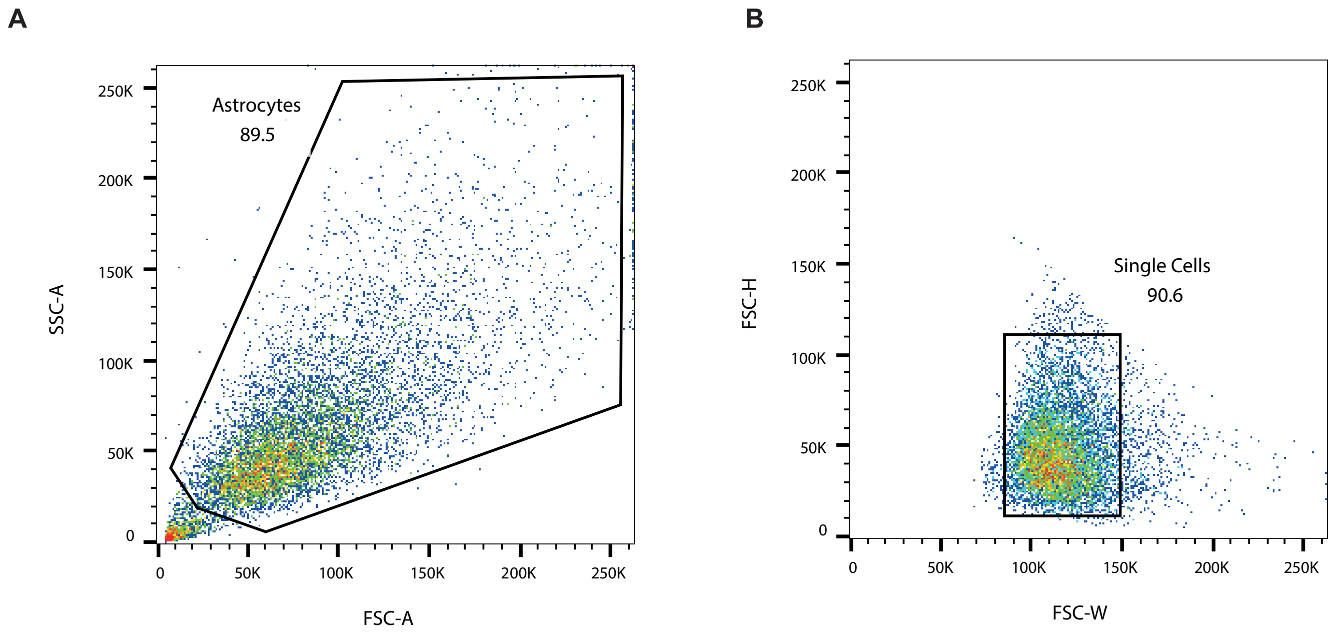

Supplementary Figure 5. Gating strategy for living single cells in Astrocyte flow cytometry experiments A) Representative pseudocolor plot of FSC-A vs SSC-A signal to gate out debris from cells in flow cytometry samples. B) Representative pseudocolor plot of FSC-W vs FSC-H signal of all cells selected with the gate in Figure A, to select single cells. Geometric mean fluorescent intensity for anti-HLA-A,B,C-PE was determined from the single cell population in all flow cytometry data presented in the main figures.

Supplementary Table 1. The Neurolipid Atlas: an open access data common for brain lipid data Description of datasets currently available on the Neurolipid Atlas.

Sample type	Experiment	Contributing lab
**iPSC-derived neurons**	1. Multiple iNeuron inductions from 3 C9orf72 lines and 3 isogenic control lines (from Giblin *et al.*)	Isaacs lab
	2. Multiple iNeuron inductions from 3 C9orf72 lines treated with C9orf72-knockdown antisense oligonucleotides (from Giblin *et al.*)	Isaacs lab
	3. Multiple iNeuron inductions from 2 control lines transduced with C9orf72 repeat lentiviruses (from Giblin *et al.*)	Isaacs lab
	4. Multiple iNeuron inductions from 3 C9orf72 lines transduced with FAT1 or FAT2 lentiviruses (from Giblin *et al.*)	Isaacs lab
	5. alpha-synuclein E46K neurons	Van der Kant lab
	6. TMEM106B SS185 vs TT185 vs Null iNeurons	Ward lab
**iPSC-derived astrocytes**	1. Multiple repeats of control vs reactive (IL1α_TNF_C1q) ApoE3 iAstrocytes in three cell lines: WTC11 Rose, BIONi037-A & Kolf2.1J (from Feringa and Koppes-den Hertog *et al.*)	Kampmann lab & Van der Kant lab
	2. Multiple repeats of control vs reactive (IL1α_TNF_C1q) ApoE4 iAstrocytes in two cell lines: BIONi037-A4 & Kolf2.1J (from Feringa and Koppes-den Hertog et al.)	Van der Kant lab
	3. Multiple repeats of ApoE4 vs ApoE3 isogenic iAstrocytes with FBS in two cell lines: BIONi037-A & Kolf2.1J	Van der Kant lab
	4. Multiple repeats of ApoE4 vs ApoE3 isogenic iAstrocytes no FBS in three cell lines: BIONi037-A, Kolf2.1J set #1 & Kolf2.1J set #2 (from Feringa and Koppes-den Hertog *et al.*)	Van der Kant lab
	5. Multiple repeats of ApoE3 iAstrocytes cultured with FBS versus no FBS in two cell lines: BIONi037-A & Kolf2.1J	Van der Kant lab
**iPSC-derived neurons, microglia and astrocytes**	1. iPSC-derived neurons, microglia and astrocytes (from Feringa and Koppes-den Hertog *et al.*)	Van der Kant lab Kronenberg-Versteeg lab
**Mouse astrocytes**	1. Control vs reactive (IL1α_TNF_C1q) mouse astrocytes (from Feringa and Koppes-den Hertog *et al.*)	Van der Kant lab
**Mouse bone marrow-derived macrophages (BMDM's)**	1. NPC1 cKO vs control mouse BMDM's (from Wang *et al.*)	Ward lab
**Human brain**	1. Human brain AD vs control in three brain regions: cerebellum, prefrontal cortex gray matter and prefrontal cortex white matter (from Feringa and Koppes-den Hertog *et al.*)	Lashley lab Van der Kant lab

	2. Human prefrontal cortex gray matter, white matter and cerebellum all control (from Feringa and Koppes-den Hertog *et al.)*	Lashley lab<br>Van der Kant lab
	3. Human brain FTLD vs control in two brain regions: cerebellum and prefrontal cortex gray matter (from Giblin *et al.*)	Lashley lab<br>Isaacs lab
**Mouse brain**	1. Mouse cortex ApoE4 vs ApoE3 target replacement mice 3 and 6 months old	Van der Kant lab
**Drosophilia brain**	1. C9orf72 Drosophilia brains (RNA only/G4C2-36/GR36 vs WT) (from Giblin *et al.*)	Isaacs lab
	2. C9orf72 Drosophilia brains (G4C2-36 + Desat1/FAT2) (from Giblin *et al.*)	Isaacs lab

Supplementary Table 2. Metadata human postmortem brain tissue

Sample nr	PM delay	AAO	AAD	Duration	Gender	Clinical Diag	Path Diag	Cleanedpat hdiag	Brain Weight	APOE	Braak Tau	Thal Phase	CERAD	ABC	CAA
1	34:00:00	54	63	9	M	Picks/FTD (bvFTD)	AD	AD	1150	44	6	5	3	A3B3C3	2
2	90:05:00	64	77	13	M	AD (Amnestic)	AD	AD	1264	44	6	5	3	A3B3C3	3
3	60:25:00	59	76	17	F	AD	AD	AD	1191	44	6	5	3	A3B3C3	2
4	70:00:00	55	68	13	M	DLB	AD	AD	1522	33	6	5	3	A3B3C3	1
5	76:40:00	49	62	13	F	AD (Amnestic)	AD	AD	996	33	6	5	3	A3B3C3	0
6	84:45:00	58	69	11	M	PPA / AD	AD	AD	1600	33	6	5	3	A3B3C3	3
7	61:19:00	63	79	16	M	AD	AD	AD	1423	33	6	5	3	A3B3C3	3
8	78:15:00	69	81	12	M	AD	AD	AD	1116	33	6	5	3	A3B3C3	2
9	31:42:00	48	63	15	M	AD	AD	AD	1042	33	6	5	3	A3B3C3	3
10	34:25:00	54	65	11	M	AD	AD	AD	1089	44	6	5	3	A3B3C3	3
11	17:10:00	55	67	12	F	PPA	AD	AD	1009	33	6	5	3	A3B3C3	3
12	14:50:00	62	72	10	M	PD	AD	AD	1180	44	6	5	3	A3B3C3	2
13	66:00:00	51	60	9	F	CBD	AD	AD	1090	33	6	5	3	A3B3C3	3
14	33:26:00	63	74	11	M	AD	AD	AD	1022	44	6	5	3	A3B3C3	3
15	44:05:00	68	84	16	F	VD	AD	AD	1127	44	6	5	3	A3B3C3	3
16	45:35:00	52	71	19	M	AD	AD	AD	1097	33	6	5	3	A3B3C3	3
17	52:30:00	53	68	15	F	Picks	AD	AD	1103	44	6	5	3	A3B3C3	1
18	40:10:00	49	69	20	F	AD	AD	AD	986	44	6	5	2	A3B3C2	3
19	92:47:00	50	66	16	F	Picks (PPA) (Amnestic)	AD	AD	906	33	6	5	3	A3B3C3	2
20	30:25:00	59	79	20	F	AD	AD	AD	961	33	6	5	3	A3B3C3	3
21	80:00:00		64		M	Control	Control	Control	1695	33	0	1	0	A1B0C0	0
22	47:00:00		73		M	Control	Control	Control	1291	33	4	1	0	A0B2C0	0
23	171:00:00		69		M	Control	Control/path aging	Control	1435	33	1	3	1	A2B1C1	0
24	26:46:00		70		F	Dystonia?	Control	Control	1200	23	1	0	0	A0B1C0	0
25	79:00:00		76		M	Control	Control	Control	1366	34	2	1	0	A1B1C0	0
26	24:00:00		73		F	Control	Path Ageing	Control	1214	34	2	2	2	A1B1C2	1
27	76:10:00		71		F	Control	Control	Control	1214	33	3	2	1	A1B1C0	2
28	47:05:00		89		M	Control	Control	Control	1356	33	2	3	1	A2B1C1	1
29	29:30:00		78		F	Control	Control age related changes	Control	1225	22	2	0	0	A0B2C0	0
30	88:50:00		79		F	Control	Control	Control	1288	33	1	2	1	A2B1C1	0
31	45:05:00		68		F	Control	Control	Control	1330	23	0	0	0	A0B0C0	0
32	54:20:00		73		M	Control	Control	Control	1498	24	3	4	2	A3B2C2	3
33	3:30:00		79		M	Control	Control	Control	1355	33	2	0	0	A0B1C0	0

Supplementary Table 3. Metadata information sheet for lipidomics data

Metadata information needed for each sample	Explanation
**Experiment title**	Name the experiment/comparison: Condition X vs condition Y
**Process date**	Lipidomics run date
**Sample number**	Assign each biological, blank and QC sample with a number 1/2/3/...n
**Reference Group**	Does this sample belong to the wild type/control group to which other samples need to be compared to? Fill in Yes or No.
**Protein quantity**	Protein quantity if measured (only when applicable, otherwise NA)
**Harvest Date**	Sample harvest date
**Sample Type**	Cell Type or human/mouse/csf material
**Genotype**	Genotype of the sample of interest for comparison (e.g. APOE4/4)
**Parental Cell line**	Parental iPSC-line name (only when applicable, otherwise NA)
**Additional Cell line sample details**	Specific name of clone (only when applicable, otherwise NA)
**Brain region**	For mouse/human brain tissue (only when applicable, otherwise NA)
**Sex**	Male or Female
**Culture conditions**	Add information on deviations in culture conditions (e.g. without serum, with supplement X, days of differentiation etc., otherwise NA)
**Drug treatment**	Indicate drug/compound cells have been treated with (only when applicable, otherwise NA)
**Diagnosis**	Clincial diagnosis in case of tissue or csf, can also be control.
**Cell count**	Cell number harvested for lipidomics (only when applicable, otherwise NA)
**Notes**	Additional information (only when applicable, otherwise NA)
**Lab**	Lab that provided the material
**Machine**	Name of lipidomics platform used

Supplementary Table 4. Primers used for qPCR

**Gene**	**Species**	**Primer sequence**	**Marker for**
PSMB9 fwd	Human	GCACCAACCGGGGACTTAC	Immunoproteosome member
PSMB9 rev	Human	CACTCGGGAATCAGAACCCAT	Immunoproteosome member
HLA-B fwd	Human	CAGTTCGTGAGGTTCGACAG	MHC I pathway
HLA-B rev	Human	CAGCCGTACATGCTCTGGA	MHC I pathway
IRF1 fwd	Human	ATGCCCATCACTCGGATGC	Ifn regulated TF
IRF1 rev	Human	CCCTGCTTTGTATCGGCCTG	Ifn regulated TF
NLRC5 fwd	Human	GCTCGGCAACAAGAACCTGT	Reg of MHC I expression
NLRC5 rev	Human	GGTCCAAGGTCTCGTTCCT	Reg of MHC I expression
B2M fwd	Human	GAGGCTATCCAGCGTACTCCA	MHC I pathway
B2M rev	Human	CGGCAGGCATACTCATCTTTT	MHC I pathway
GAPDH fwd	Human	GTCTCCTCTGACTTCAACAGCG	Housekeeping gene
GAPDH rev	Human	ACCACCCTGTTGCTGTAGCCAA	Housekeeping gene
IRF1 fwd	Mouse	ATGCCAATCACTCGAATGCG	Ifn regulated TF
IRF1 rev	Mouse	CCTGCTTTGTATCGGCCTGT	Ifn regulated TF
H2-D1 fwd	Mouse	TCCGAGATTGTAAAGCGTGAAGA	MHC I pathway
H2-D1 rev	Mouse	ACAGGGCAGTGCAGGGATAG	MHC I pathway
B2M fwd	Mouse	TTCTGGTGCTTGTCTCACTGA	MHC I pathway
B2M rev	Mouse	CAGTATGTTCGGCTTCCCATTC	MHC I pathway
GAPDH fwd	Mouse	CATCACTGCCACCCAGAAGACTG	Housekeeping gene
GAPDH rev	Mouse	ATGCCAGTGAGCTTCCCGTTCAG	Housekeeping gene
		**qPCR primers used for iMicroglia:**	
PPIB fwd	Human	CACAGGAGGAAAGAGCATCTAC	Housekeeping gene
PPIB rev	Human	CTGTCTTGACTGTCGTGATGAA	Housekeeping gene
RPLP0 fwd	Human	GCCATTGCCCCATGTGAAGT	Housekeeping gene
RPLP0 rev	Human	AGCTGCACATCACTCAGGATT	Housekeeping gene
ITGAM fwd	Human	TCTTTGCTTTGGTGGCTTCCT	CD11b – microglia marker
ITGAM rev	Human	TGCGTTTTCAGTGTCCAAGTT	CD11b – microglia marker
CX3CR1 fwd	Human	TGTGACTGAGACGGTTGCAT	Homeostatic microglia
CX3CR1 rev	Human	TTCCATGCCTGCTCCTTTGT	Homeostatic microglia
SPI1 fwd	Human	TTGAAAAAGGAGTTGGTGGC	PU.1 – microglial TF
SPI1 rev	Human	TGCTGGTTCTGTAAGTTGGG	PU.1 – microglial TF
TMEM119 fwd	Human	AGCACGGACTCTCTCTTCCA	Homeostatic microglia
TMEM119 rev	Human	GACCAGTTCCTTGGCGTACA	Homeostatic microglia
P2RY12 fwd	Human	TGCCAAACTGGGAACAGGACCA	Homeostatic microglia
P2RY12 rev	Human	TGGTGGTCTTCTGGTAGCGATC	Homeostatic microglia
