# Supplementary material for: The Neurolipid Atlas: a lipidomics resource for neurodegenerative diseases
Source: Nat Metab. 2025 Sep 22;7(10):2142–64. doi: 10.1038/s42255-025-01365-z (PMC12552125; doi:10.1038/s42255-025-01365-z)
Supplement: Supplementary file 2 — Reporting Summary [file 42255_2025_1365_MOESM2_ESM.pdf]

nature portfolio

Corresponding author(s): Rik van der Kant
R.h.n.vander.kant@vu.nl

Last updated by author(s): Jul 30, 2025

Reporting Summary

Nature Portfolio wishes to improve the reproducibility of the work that we publish. This form provides structure for consistency and transparency in reporting. For further information on Nature Portfolio policies, see our Editorial Policies and the Editorial Policy Checklist.

Statistics

For all statistical analyses, confirm that the following items are present in the figure legend, table legend, main text, or Methods section.

n/a	Confirmed	
☐	☒	The exact sample size (n) for each experimental group/condition, given as a discrete number and unit of measurement
☐	☒	A statement on whether measurements were taken from distinct samples or whether the same sample was measured repeatedly
☐	☒	The statistical test(s) used AND whether they are one- or two-sided<br>*Only common tests should be described solely by name; describe more complex techniques in the Methods section.*
☐	☒	A description of all covariates tested
☐	☒	A description of any assumptions or corrections, such as tests of normality and adjustment for multiple comparisons
☐	☒	A full description of the statistical parameters including central tendency (e.g. means) or other basic estimates (e.g. regression coefficient) AND variation (e.g. standard deviation) or associated estimates of uncertainty (e.g. confidence intervals)
☐	☒	For null hypothesis testing, the test statistic (e.g. F, t, r) with confidence intervals, effect sizes, degrees of freedom and P value noted<br>*Give P values as exact values whenever suitable.*
☒	☐	For Bayesian analysis, information on the choice of priors and Markov chain Monte Carlo settings
☒	☐	For hierarchical and complex designs, identification of the appropriate level for tests and full reporting of outcomes
☒	☐	Estimates of effect sizes (e.g. Cohen's d, Pearson's r), indicating how they were calculated

Our web collection on statistics for biologists contains articles on many of the points above.

Software and code

Policy information about availability of computer code

Data collection	Sony SH800S Cell Sorter (Sony Biotechnologies, San Jose, California) was used to acquire flow cytometry data of the iMicroglia.<br>BD LSRFortessa X-20 (BD Biosciences, Franklin Lakes, NJ, USA) was used to acquire flow cytometry data of the iAstrocytes.<br>Fluorescent images were collected on the Cell Insight CX7 LED Pro HCS Platform (Fisher Scientific, Hampton, NH, USA) or on a Nikon Ti-Eclipse microscope (Amstelveen, The Netherlands).<br>qPCR data was acquired with QuantStudio 3 RT PCR system (Thermo Fisher Scientific).<br>Western blot images were taken with LI-COR® Odyssey® Fc Imaging System (LI-COR, Cambridge, UK).
Data analysis	(Statistical) analyses were performed in Graphpad Prism version 10.2.3 (GraphPad Software, Boston, MA, USA) or Rstudio version 2023.9.1 (RStudio, PBC, Boston, MA, USA).<br>Flow cytometric analysis was done in FlowJo™ (BD Biosciences, Franklin Lakes, NJ, USA).<br>Fluorescent image analysis was done using Columbus® version 2.5.2 (PerkinElmer, Waltham, MA, USA) or Fiji (Schindelin et al., 2012).<br>Western blot analysis was done using Image Studio™ Lite 5.2.5 Software (LI-COR, Cambridge, UK).<br>Webgestalt open access analysis platform was used for GSEA and ORA analyses. The version used: https://2019.webgestalt.org/<br>RNAseq data from TCW et al. 2022 and Lin et al. 2018 was downloaded and reanalyzed using GEO2R and Rstudio version 2023.9.1 (RStudio, PBC, Boston, MA, USA).

For manuscripts utilizing custom algorithms or software that are central to the research but not yet described in published literature, software must be made available to editors and reviewers. We strongly encourage code deposition in a community repository (e.g. GitHub). See the Nature Portfolio guidelines for submitting code & software for further information.

Data

Policy information about availability of data

All manuscripts must include a data availability statement. This statement should provide the following information, where applicable:

- Accession codes, unique identifiers, or web links for publicly available datasets
- A description of any restrictions on data availability
- For clinical datasets or third party data, please ensure that the statement adheres to our policy

Sequencing data is deposited in the Gene Expression Omnibus repository. Bulk RNA-seq of ApoE3/3 and ApoE4/4 iPSC-derived astrocytes from the BIONi037-A and KOLF2.1J lines is under accession code GSE302826. All proteomics data is deposited on MassIVE. Proteomics data of ApoE3/3 and ApoE4/4 iPSC-derived astrocytes from the BIONi037-A line is under accession code MSV000098665 and from the KOLF2.1J line is under accession code MSV000098666. Proteomics data of control and reactive iPSC-derived astrocytes from the BIONi037-A and KOLF2.1J lines is under accession code MSV000098668. Lipidomics data is available through www.neurolipidatlas.com. Uncropped western blots can be found in source data.

Research involving human participants, their data, or biological material

Policy information about studies with human participants or human data. See also policy information about sex, gender (identity/presentation), and sexual orientation and race, ethnicity and racism.

Reporting on sex and gender	For the lipidomics analysis of postmortem control vs AD tissue all metadata including sex is available in Supplementary Table 2. For all iPSC-derived brain cell types sex is also described in the methods, figures and figure legends.
Reporting on race, ethnicity, or other socially relevant groupings	N/A
Population characteristics	All populations characteristics can be found in Supplementary Table 2.
Recruitment	N/A
Ethics oversight	All research complies with the relevant ethical regulations and institutional guidelines at the Vrije Universiteit in Amsterdam. For postmortem tissue: Ethical approval for the study was obtained from the NHS research ethics committee (NEC) and in accordance with the human tissue authority's (HTA's) code of practice and standards under licence number 12198.

Note that full information on the approval of the study protocol must also be provided in the manuscript.

Field-specific reporting

Please select the one below that is the best fit for your research. If you are not sure, read the appropriate sections before making your selection.

☒ Life sciences ☐ Behavioural & social sciences ☐ Ecological, evolutionary & environmental sciences

For a reference copy of the document with all sections, see nature.com/documents/nr-reporting-summary-flat.pdf

Life sciences study design

All studies must disclose on these points even when the disclosure is negative.

Sample size	All sample sizes and independent number of experiments are stated in the figure legends. All iPSC-derived astrocyte experiments were performed on two independent iPSC-background lines with isogenic ApoE3/3 or ApoE4/4 variants. Sample sizes were chosen based on comparable previous studies.
Data exclusions	One BIONI037 APOE4 astrocyte sample from lipidomics E4 vs E3 analysis I (noFBS) was excluded as outlier. In figure 6J one ourlier experiment was removed based on ROUT (Q=1).
Replication	All replication experiments are reported in the manuscript. All data was replicated in two independent iPSC-background lines.
Randomization	For all lipidomics data, the order of samples was randomized before each batch was run on the Lipidyzer platform.
Blinding	All data analysis was done by automated quantatative measurments with no bias involvement, therefore blinding was not necessary for this study.

Reporting for specific materials, systems and methods

We require information from authors about some types of materials, experimental systems and methods used in many studies. Here, indicate whether each material, system or method listed is relevant to your study. If you are not sure if a list item applies to your research, read the appropriate section before selecting a response.

Materials & experimental systems

n/a	Involved in the study
☐	☒ Antibodies
☐	☒ Eukaryotic cell lines
☒	☐ Palaeontology and archaeology
☐	☒ Animals and other organisms
☒	☐ Clinical data
☒	☐ Dual use research of concern
☒	☐ Plants

Methods

n/a	Involved in the study
☒	☐ ChIP-seq
☐	☒ Flow cytometry
☒	☐ MRI-based neuroimaging

Antibodies

Antibodies used

APC anti-human CD45 Antibody, Mouse IgG1, HI30 (BioLegend, 304011), Brilliant Violet 421™ anti-mouse/human CD11b Antibody, Clone M1/70, Rat IgG2b (Biolegend, 101235), anti-perilipin 2 (15294-1-AP, Proteintech), anti-AQP4 (AQP-004, Alomone labs), anti-GFAP (173 004, Synaptic systems), anti-Vimentin (sc-6260, Santa Cruz Biotechnology), anti-MAP2 (ab5392, Abcam), anti-Smi 312 (SMI-312P-050, Eurogentec), anti-Iba1 (NB100-1028, Novus Biologicals), anti-PU.1 (MA5-15064, Thermo Fisher Scientific) anti-HLA Class I Heavy Chain (kind gift from Prof. Dr. Neefjes), PE anti-human HLA-A,B,C Antibody (BioLegend, 311406), anti-GAPDH (elabscience, E-AB-40337), anti-TAP1 (kind gift from Prof. Dr. Neefjes), anti-TAP2 (kind gift from Prof. Dr. Neefjes)

Validation

All, but three, antibodies are commercially available and have been validated by the manufacturers and in previous publications. All information can be found on the manufacturers' websites.
APC anti-human CD45 Antibody, Mouse IgG1, HI30: https://www.biolegend.com/nl-nl/products/apc-anti-human-cd45-antibody-705
Brilliant Violet 421™ anti-mouse/human CD11b Antibody, Clone M1/70, Rat IgG2b: https://www.biolegend.com/nl-nl/products/brilliant-violet-421-anti-mouse-human-cd11b-antibody-7163
anti-perilipin 2 https://www.ptglab.com/products/ADRP-Antibody-15294-1-AP.htm
anti-AQP4: https://www.alomone.com/p/anti-aquaporin-4-antibody/AQP-004
anti-GFAP: https://sysy.com/product/173004
anti-Vimentin: https://www.scbt.com/p/vimentin-antibody-v9
anti-MAP2: https://www.abcam.com/en-us/products/primary-antibodies/map2-antibody-neuronal-marker-ab5392
anti-Smi 312: SMI-312P-050, Eurogentec
anti-Iba1: https://www.novusbio.com/products/aif-1-iba1-antibody_nb100-1028
anti-PU.1: https://www.thermofisher.com/antibody/product/PU-1-Antibody-clone-E-388-3-Monoclonal/MA5-15064
PE anti-human HLA-A,B,C Antibody: https://www.biolegend.com/nl-nl/products/pe-anti-human-hla-a-b-c-antibody-1872
anti-GAPDH: https://www.elabscience.com/p/gapdh-polyclonal-antibody--e-ab-40337

Three antibodies were a kind gift from Prof. Dr. Neefjes
anti-HLA Class I Heavy Chain: From HCA2 clone generated by hybridoma. This clone is also commercially available and has been validated in Stam et al. 1990 International Immunology
anti-TAP1: Made in-house. Described in https://doi.org/10.1002/eji.1830180522
anti-TAP2: Made in-house. Described in https://doi.org/10.1002/eji.1830180522

Eukaryotic cell lines

Policy information about cell lines and Sex and Gender in Research

Cell line source(s)

Isogenic Kolf2.1J (APOE3/3), Kolf2.1J C112R Hom3 (APOE4/4 set #1) and Kolf2.1J C112R Hom2 (APOE4/4 set #2) human iPSCs were a kind gift from INDi (Donor 57y male). Kolf2.1J C112R Hom2 (APOE4/4 set #2) corresponds to JIPSC001142 available via INDI. Isogenic BIONi037-A (APOE3/3) and BIONi037-A4 (APOE4/4) human iPSC lines were obtained via EBISC (Donor 77y female).

Authentication

The identify of the cell lines was verified by the supplier and we authenticated the cell lines based on morphology.

Mycoplasma contamination

Cell cultures were regularly tested for mycoplasma contamination. No mycoplasma contamination was detected.

Commonly misidentified lines
(See ICLAC register)

N/A

Animals and other research organisms

Policy information about studies involving animals; ARRIVE guidelines recommended for reporting animal research, and Sex and Gender in Research

Laboratory animals

Timed-pregnant C57bl6j mice were purchased from Charles River (Wilmington, MA) or bred in house. Brains were extracted from

Laboratory animals	both male and female pups (E18-P3). Cortices were isolated after meninges removal.
Wild animals	N/A
Reporting on sex	Both male and female pups were used.
Field-collected samples	N/A
Ethics oversight	All research complies with the relevant ethical regulations and institutional guidelines at the Vrije Universiteit in Amsterdam.

Note that full information on the approval of the study protocol must also be provided in the manuscript.

Plants

Seed stocks	N/A
Novel plant genotypes	N/A
Authentication	N/A

Flow Cytometry

Plots

Confirm that:

☒ The axis labels state the marker and fluorochrome used (e.g. CD4-FITC).

☒ The axis scales are clearly visible. Include numbers along axes only for bottom left plot of group (a 'group' is an analysis of identical markers).

☒ All plots are contour plots with outliers or pseudocolor plots.

☒ A numerical value for number of cells or percentage (with statistics) is provided.

Methodology

Sample preparation	After accutase dissocation, FACS buffer, DPBS + 2% FBS (Fisher Scientific, A5256701), was added to a volume of max. 300 µL and the iAstrocytes were transferred to a round bottom 96 well plate. After centrifuging for 1 minute at 2000 RPM, the iAstrocytes were stained with 1:50 PE anti-human HLA-A,B,C Antibody (BioLegend, 311406) for 30 minutes at 4°C. Following another centrifugation step, the iAstrocytes were fixed with 2% formaldehyde (Sigma Aldrich, P6148) for 15 minutes at RT.
Instrument	BD LSRFortessa X-20 (BD Biosciences, Franklin Lakes, NJ, USA)
Software	Data analysis was done in FlowJo™ (BD Biosciences, Franklin Lakes, NJ, USA)
Cell population abundance	Live cell population was determined by FSC-A vs SSC-A, where 80-90% of total events fell within the gate for each sample. Single cell population was determined by FSC-W vs FSC-H on the live gated cells. For each sample 80-90% of cells were selected based on the single cell gate.
Gating strategy	Live and single cells were gated out as described above. For each sample the geometric mean fluorescent intensity for the HLA-A,B,C antibody stain was determined within the single cell population.

☒ Tick this box to confirm that a figure exemplifying the gating strategy is provided in the Supplementary Information.
