# Supplementary material for: The Neurolipid Atlas: a lipidomics resource for neurodegenerative diseases
Source: Nat Metab. 2025 Sep 22;7(10):2142–64. doi: 10.1038/s42255-025-01365-z (PMC12552125; doi:10.1038/s42255-025-01365-z)

Fig. 4H

HLA Class I Heavy Chain: membrane was cut between 55 and 25 on the left and 35 on the right. Samples on the right are from a different experiment.

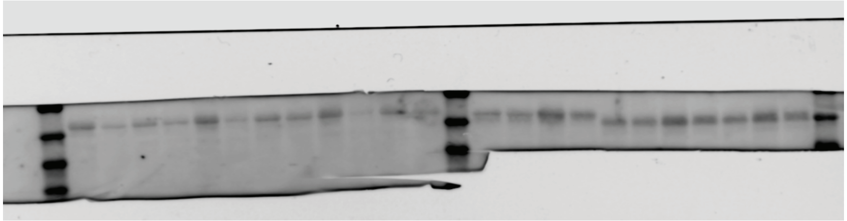

GAPDH: membrane was cut between 55 and 25 on the left and 35 on the right. Samples on the right are from a different experiment.

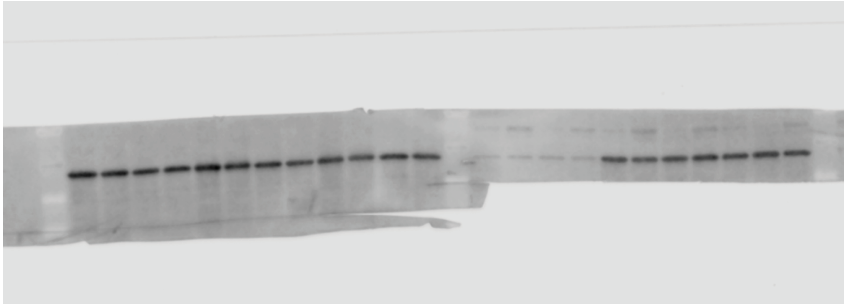

Supplement: Supplementary file 3 — Unprocessed western blots. [file 42255_2025_1365_MOESM3_ESM.pdf]
