# Supplementary material for: The Neurolipid Atlas: a lipidomics resource for neurodegenerative diseases
Source: Nat Metab. 2025 Sep 22;7(10):2142–64. doi: 10.1038/s42255-025-01365-z (PMC12552125; doi:10.1038/s42255-025-01365-z)

# Extended data fig. 5C

TAP1: membrane was cut at 55 kDa.

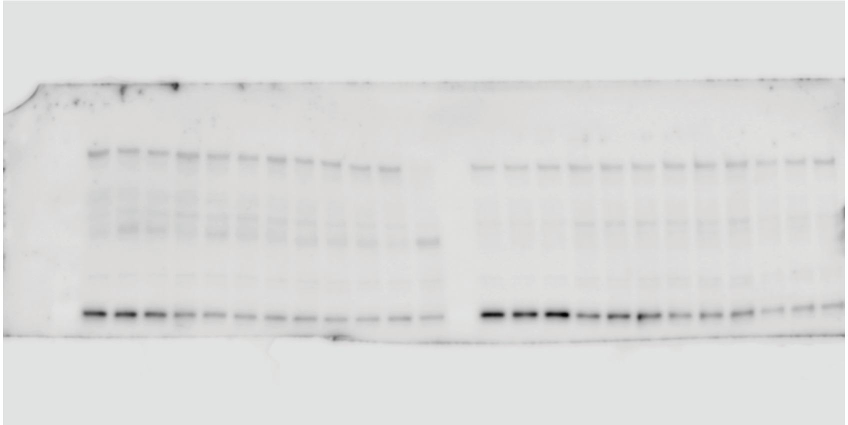

GAPDH: The membrane was cut at 55 kDa and between 25 and 15 kDa.

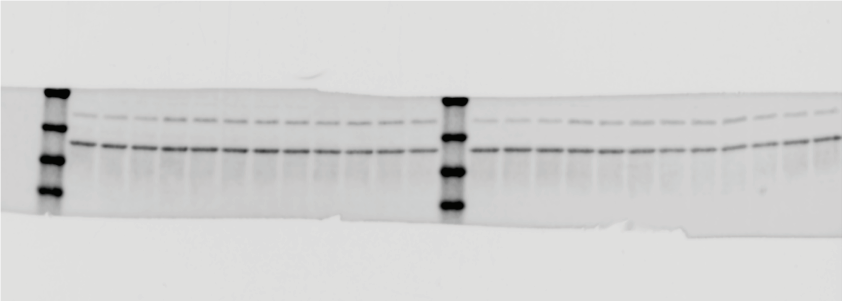

TAP2: The empty space at the top of the membrane was cut off and at the bottom the membrane was cut at 55 kDa.

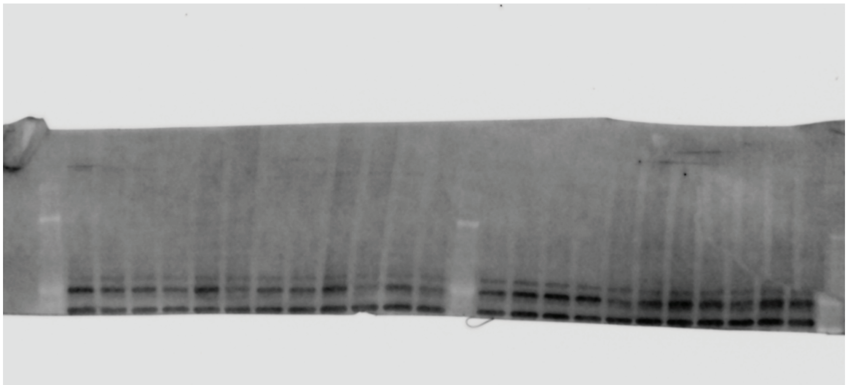

GAPDH: membrane was cut between 55 kDa and 25 kDa on the left and 35 kDa on the right. Samples on the right are from a different experiment.

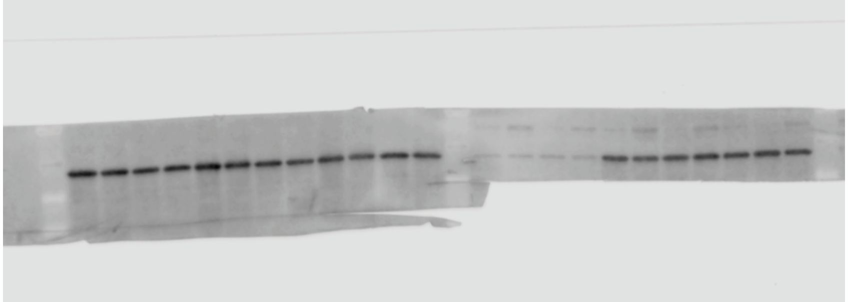

Supplement: Supplementary file 4 — Unprocessed western blots. [file 42255_2025_1365_MOESM4_ESM.pdf]
